# Supplementary material for: Polθ inhibitors elicit BRCA-gene synthetic lethality and target PARP inhibitor resistance
Source: Nat Commun. 2021 Jun 17;12:3636. doi: 10.1038/s41467-021-23463-8 (PMC8211653; doi:10.1038/s41467-021-23463-8)
Supplement: Supplementary file 1 — Supplementary Information [file 41467_2021_23463_MOESM1_ESM.pdf]

Supplementary Materials for:

# Polθ inhibitors elicit *BRCA*-gene synthetic lethality and target PARP-inhibitor resistance

Diana Zatreanu[1,2]†, Helen M.R. Robinson[3]†, Omar Alkhatib[3], Marie Boursier[3], Harry Finch[3], Lerin Geo[3], Diego Grande[3], Vera Grinkevich[3], Robert A. Heald[3], Sophie Langdon[3], Jayesh Majithiya[3], Claire McWhirter[3], Niall M.B. Martin[3], Shaun Moore[3], Joana Neves[3], Eeson Rajendra[3], Marco Ranzani[3], Theresia Schaedler[3], Martin Stockley[3], Kimberley Wiggins[3], Rachel Brough[1,2], Sandhya Sridhar[1,2], Aditi Gulati[1,2], Nan Shao[1,2], Luned M. Badder[4], Daniela Novo[2], Eleanor G. Knight[2], Rebecca Marlow[2,4], Syed Haider[2], Elsa Callen[5], Graeme Hewitt[6], Joost Schimmel[7], Remko Prevo[8], Christina Alli[9], Amanda Ferdinand[9], Cameron Bell[9], Peter Blencowe[9], Chris Bot[9], Mathew Calder[9], Mark Charles[9], Jayne Curry[9], Tennyson Ekwuru[9], Katherine Ewings[9], Wojciech Krajewski[9], Ellen MacDonald[9], Hollie McCarron[9], Leon Pang[9], Chris Pedder[9], Laurent Rigoreau[9], Martin Swarbrick[9], Ed Wheatley[9], Simon Willis[9], Ai Ching Wong[9], Andre Nussenzweig[5], Marcel Tijsterman[7], Andrew Tutt[2,4], Simon J. Boulton[3,6], Geoff S. Higgins[8], Stephen J. Pettitt[1,2]*, Graeme C.M. Smith[3]*, Christopher J. Lord[1,2]*

**This PDF file includes:**

Supplementary Materials and Methods
Supplementary Figures 1-4
Supplementary Tables 1-4

## Supplementary Materials and Methods

**Abbreviations**
DCM, Dichloromethane
DIPEA, N,N-Diisopropylethylamine
DMF, Dimethylformamide
DMSO, Dimethylsulfoxide
EEDQ, N-Ethoxycarbonyl-2-ethoxy-1,2-dihydroquinoline
EtOAc, Ethyl acetate
HPLC, High-performance liquid chromatography
NMP, N-Methylpyrrolidinone
NMR, Nuclear magnetic resonance
PE, Petroleum ether
RT Room (or ambient) temperature
SCX-2, Propylsulfonic acid (non-endcapped) functionalized silica column
TBAF, Tetra-n-butylammonium fluoride
TBS, tert-Butyldimethylsilyl (in synthesis); Tris Buffered Saline (in molecular biology)
TFA, Trifluoroacetic acid
THF, Tetrahydrofuran

**Synthetic route to ART558: (2S,3R)-1-(3-Cyano-6-methyl-4-(trifluoromethyl)pyridin-2-yl)-3-hydroxy-N-methyl-N-(m-tolyl)pyrrolidine-2-carboxamide**

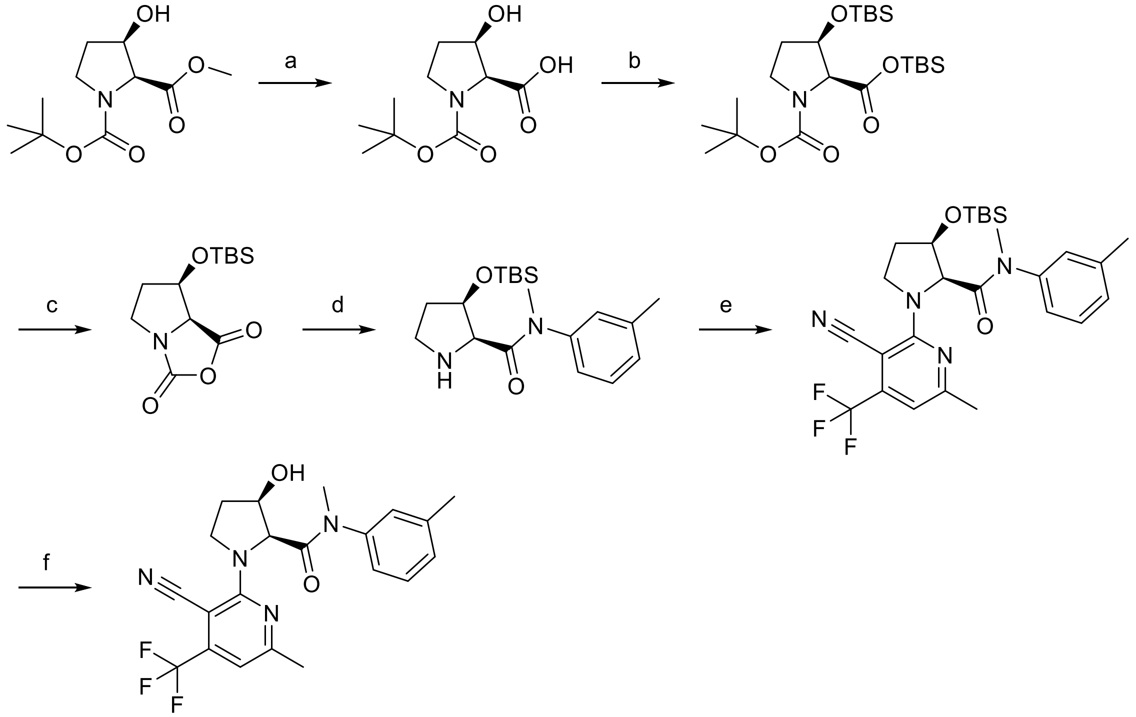


a) 1M aq. LiOH, THF, rt, 12h; b) TBSCl, imid., DMF, rt, 15h; c) oxalyl chloride, cat. DMF, DCM, 0-15°C, 30 min; d) N-methyl-3-methyl-aniline, DMF, 90°C, 15h; e) 2-chloro-6-methyl-4-(trifluoromethyl)-pyridine-3-carbonitrile, DIPEA, NMP, 100°C, 2h; f) 1M TBAF, THF, 50°C, 3h.

**Step a.** To a solution of 1-(tert-butyl) 2-methyl (2S,3R)-3-hydroxypyrrolidine-1,2-dicarboxylate (CAS Number 186132-96-7; 10.0 g, 40.8 mmol) in THF (50 mL) was added a 1M aqueous solution of lithium hydroxide (61 mL, 61 mmol) at rt and the mixture was stirred for 12 h. The reaction mixture was extracted with EtOAc (50 mL x 3). The aqueous layer was acidified with 1M HCl to pH 6 and then evaporated *in vacuo*. MeOH (100 mL) was added to the residue, stirred at RT for 10 mins and the resulting suspension was filtered. The filtrate was concentrated under reduced pressure to give (2S,3R)-1-(tert-butoxycarbonyl)-3-hydroxypyrrolidine-2-carboxylic acid (10.5 g, crude) as a white solid.

$^1$H NMR (400 MHz, $D_2O$) δ ppm 4.58 - 4.54 (m, 1H), 4.19 - 4.15 (m, 1H), 3.58 - 3.41 (m, 2H), 2.09 - 2.01 (m, 1H), 1.91 - 1.86 (m, 1H), 1.44 - 1.39 (m, 9 H).

**Step b.** To the suspension of (2S,3R)-1-(tert-butoxycarbonyl)-3-hydroxypyrrolidine-2-carboxylic acid (4.00 g, 17.3 mmol) in DMF (40 mL) was added TBSCl (10.4 g, 69.2 mmol) and imidazole (7.07 g, 104 mmol) at rt and the mixture was stirred for 15 h. The reaction mixture was diluted with water (300 mL) and extracted with EtOAc (200 mL x 3). The combined organic layers were washed with brine (200 mL x 2), dried over $Na_2SO_4$, filtered and evaporated *in vacuo*. The residue was purified by column chromatography, eluting with 10-30% EtOAc in PE. Product fractions were combined and solvent was removed *in vacuo* to give 1-(tert-butyl)-2-(tert-butyldimethylsilyl)-(2S,3R)-3-((tert-butyldimethylsilyl)oxy)-pyrrolidine-1,2-dicarboxylate (6.94 g, crude) as a colorless oil.

$^1$H NMR (400MHz, $CDCl_3$) δ ppm 4.51 (q, J = 8.0 Hz, 1H), 4.29 - 4.18 (m, 1H), 3.68 - 3.59 (m, 1H), 3.36 - 3.28 (m, 1H), 2.14 - 2.02 (m, 2H), 1.44 - 1.43 (m, 9 H), 0.94 (s, 9H), 0.89 - 0.88 (m, 9H), 0.30 - 0.26 (m, 6H), 0.10 - 0.08 (m, 6H).

**Step c.** To the stirred suspension of 1-(tert-butyl)-2-(tert-butyldimethylsilyl)(2S,3R)-3-((tert-butyldimethylsilyl)oxy)pyrrolidine-1,2-dicarboxylate (6.94 g, 15.1 mmol) in DCM (70 mL) was added oxalyl chloride (1.98 mL, 22.6 mmol) and DMF (0.35 mL, 4.54 mmol) at 0°C and the mixture was stirred at 0 to 10°C for 30 min. The reaction mixture was evaporated *in vacuo* to give (7R,7aS)-7-((tert-butyldimethylsilyl)oxy)tetrahydro-1H,3H-

pyrrolo[1,2-c]oxazole-1,3-dione (4.10 g, crude) as a light yellow oil which was used in the subsequent step without purification.

**Step d.** To the stirred solution of (7R,7aS)-7-((tert-butyldimethylsilyl)oxy)tetrahydro-1H,3H-pyrrolo[1,2-c]oxazole-1,3-dione (crude; 4.10 g, 15.1 mmol) in DMF (41 mL) was added N-methyl-3-methyl-aniline (2.29 g, 18.9 mmol) and N-methyl-3-methyl-aniline hydrochloride (2.98 g, 18.9 mmol) at rt. The mixture was stirred at 90°C for 15 h. The mixture was cooled and evaporated *in vacuo* to give (2S,3R)-3-((tert-butyldimethylsilyl)oxy)-N-methyl-N-(m-tolyl)pyrrolidine-2-carboxamide (5.27 g, crude) as a brown oil.

m/z ES+ $[M+H]^+$ 349.2

**Steps e, f.** A mixture of (2S,3R)-3-((tert-butyldimethylsilyl)oxy)-N-methyl-N-(m-tolyl)pyrrolidine-2-carboxamide (5.27 g, 5.53 mmol), 2-chloro-6-methyl-4-(trifluoromethyl)pyridine-3-carbonitrile (CAS Number 13600-48-1; 1.41 g, 6.39 mmol) and DIPEA (3.86 mL, 22.1 mmol) in NMP (50 mL) was stirred at 100°C for 2 h. The reaction mixture was cooled to rt, treated with a 1M solution of TBAF in THF (15 mL, 15 mmol) and the mixture was stirred at 50°C for 1 h. Further TBAF solution (40 mL, 40 mmol) was added and the mixture was stirred at 50°C for 2 h. Water (100 mL) was added to the reaction mixture and extracted with EtOAc (100 mL x 3). The combined organic layers were washed with brine (100 mL x 2), dried over $Na_2SO_4$, filtered and evaporated *in vacuo*. The residue was purified by column chromatography, eluting with 2-10% EtOAc in PE, followed by mass-directed preparative HPLC. Product fractions were combined and solvent was removed *in vacuo*. The product was lyophilised to give the title compound (721 mg, 11.3% yield over 5 steps).

m/z ES+ $[M+H]^+$ 419.1; $^1H$ NMR (400 MHz, $CD_3OD$) δ ppm 7.43 - 7.37 (m, 3H), 7.26 - 7.24 (m, 1H), 6.94 (s, 1H), 4.86 (s, 1H), 4.23 - 4.16 (m, 2H), 3.97 - 3.92 (m, 1H), 3.26 (s, 3H), 2.56 (s, 3H), 2.40 (s, 3H), 2.08 - 1.92 (m, 2H); $^{19}F$ NMR (400 MHz, $CD_3OD$) δ ppm -65.464.

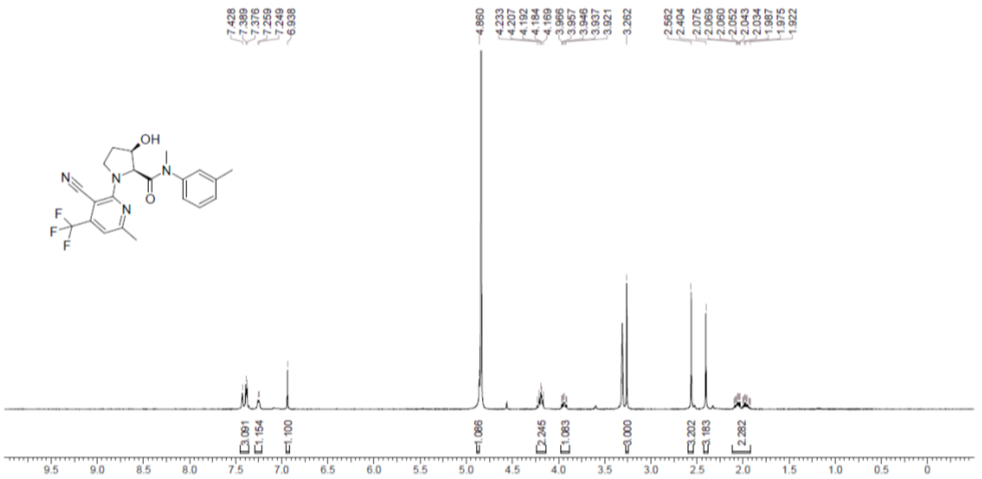

**ART558 optical rotation**
Specific Optical rotation +176.674° (Condition: C=0.4g/100mL EtOH at 20℃)

**ART558 $^{13}$C NMR**
$^{13}$C NMR (101 MHz, DMSO-d6) δ ppm 167.62, 163.73, 157.57, 143.79, 142.19 (q), 139.36, 129.69, 128.83, 124.99, 123.82, 121.09, 115.94, 109.94, 81.64, 70.06, 65.16, 48.66, 37.78, 34.29, 25.29, 21.35

**ART558 Chiral analysis**
Column: Chiralpak IC-3 50×4.6mm I.D., 3 μm
Mobile phase: Phase A for CO2, and Phase B for MeOH (0.05%DEA);
Gradient elution: MeOH (0.05% DEA) in CO2 from 5% to 40%
Flow rate: 3mL/min;Detector: PDA
Column Temp: 35C; Back Pressure: 100Bar

**Synthetic route to ART615: (2R,4R)-1-(3-Cyano-6-methyl-4-(trifluoromethyl)pyridin-2-yl)-4-hydroxy-N-methyl-N-(m-tolyl)pyrrolidine-2-carboxamide**

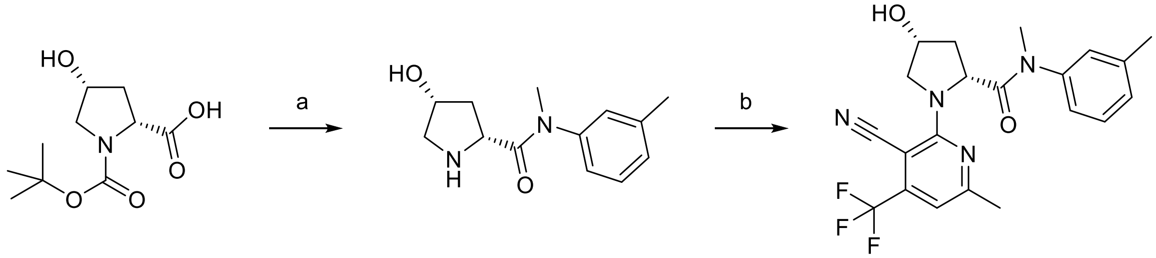

a) i. N-Methyl-3-methyl-aniline, EEDQ, DCM, rt, 15h; ii. TFA, DCM, rt, 15h; b) 2-chloro-6-methyl-4-(trifluoromethyl)-pyridine-3-carbonitrile, DIPEA, NMP, 100°C, 1h.

**Step a.** To a solution of (2R,4R)-1-(tert-butoxycarbonyl)-4-hydroxypyrrolidine-2-carboxylic acid (CAS Number 135042-12-5; 475 mg, 2.05 mmol) in DCM (10 mL) was

added N-methyl-3-methyl-aniline (0.52 mL, 4.12 mmol) and EEDQ (1.03 g, 4.12 mmol) and the mixture was stirred at rt overnight. The reaction mixture was washed with 2 M aq. HCl (2 x 25 mL), the organic layer was dried and evaporated *in vacuo*. The material was purified by column chromatography, eluting with 0-6% 7M methanolic ammonia in DCM. Product fractions were combined and solvent was removed *in vacuo*. The intermediate was re-dissolved in DCM (4 mL), treated with TFA (2.0 mL, 3.9 mmol) and stirred at RT overnight. The reaction mixture was absorbed onto SCX-2, washed with MeOH, and eluted with 7M methanolic ammonia. The solvent was removed *in vacuo* and the crude material was purified by column chromatography, eluting with 0-6% 7 M methanolic ammonia in DCM to provide (2R,4R)-4-hydroxy-N-methyl-N-(m-tolyl)pyrrolidine-2-carboxamide, (141 mg, 29% yield).

**Step b.** To a solution of (2R,4R)-4-hydroxy-N-methyl-N-(m-tolyl)pyrrolidine-2-carboxamide (70 mg, 0.30 mmol) in NMP (1.4 mL) was added 2-chloro-6-methyl-4-(trifluoromethyl)-pyridine-3-carbonitrile (CAS Number 13600-48-1; 76 mg, 0.33 mmol) and DIPEA (0.16 mL, 0.90 mmol). The mixture was heated to 100°C and stirred for 1 h. The mixture was cooled, diluted with EtOAc and washed with sat. aq. $NaHCO_3$. The organic phase was washed with brine, dried and solvent was removed *in vacuo*. The crude material was purified by mass-directed preparative HPLC. Product fractions were combined and solvent was removed *in vacuo* to provide the title compound (83 mg, 66% yield).

m/z ES+ $[M+H]^+$ 419.45; $^1$H NMR (400 MHz, DMSO-d6) δ ppm 7.40 – 7.29 (m, 1H), 7.23 – 7.07 (m, 3H), 4.67 – 4.56 (m, 1H), 3.99 (s, 1H), 3.16 (s, 3H), 2.80 – 2.73 (m, 1H), 2.45 – 2.38 (m, 1H), 2.33 (s, 3H), 1.74 – 1.56 (m, 1H), 1.49 – 1.34 (m, 1H); $^{13}$C NMR (101 MHz, DMSO-d6) δ ppm 170.80, 163.37, 156.48, 142.81, 141.77 (q), 141.61, 141.30, 139.26, 129.51, 128.71, 124.42, 123.31, 120.57, 115.37, 109.15, 81.38, 68.50, 58.50, 56.71, 37.26, 36.85, 24.84, 20.81.

Enantiopurity was confirmed as >95% ee by chiral HPLC using the enantiomer of ART615 prepared using the same synthetic method as a control.

**ART615 and ART490 chiral analysis**

ART490 Chemical purity : 100% , m/z = 419.23
Enantiomeric excess : >99.9 in favour of the second eluting isomer.

ART615 Chemical purity : 100% , m/z = 419.24
Enantiomeric excess : >99.9 in favour of the first eluting isomer.

Method: ART490 and ART615 were dissolved to ~1 mg/mL in EtOH. Aliquots of each isomer were taken, combined and then analysed by SFC and HPLC as CT176_racemate. Each individual isomer was then analysed on the same conditions, which are described in the table below.

Chiral Purity Analysis Conditions:
Column Details Lux C2 (4.6mm x 250mm, 5um)
Column Temperature 40°C
Flow Rate 4 mL/min
Detector Wavelength 210-400nm
Injection Volume 1.0 μL
BPR 125 BarG
Isocratic Conditions 50:50 MeOH:CO2 (0.2% (v/v) NH3)

Chemical Purity Analysis Conditions;
Column Details Acquity BEH C18 (50 x 2.1 mm, 1.7 μm)
Column Temperature 60°C
Flow Rate 1.0 mL/minute
Detector Wavelength 220-300nm
Injection Volume 1.0 uL
Mobile Phase A Water (0.1% v/v TFA)
Mobile Phase B MeCN
Gradient Profile Time(mins) 4 mins from 2-98 % A

**Synthetic route to ART812: (2S,3R)-1-(3-Cyano-6-methyl-4-(trifluoromethyl)pyridin-2-yl)-3-hydroxy-N-methyl-N-(m-tolyl)pyrrolidine-2-carboxamide**

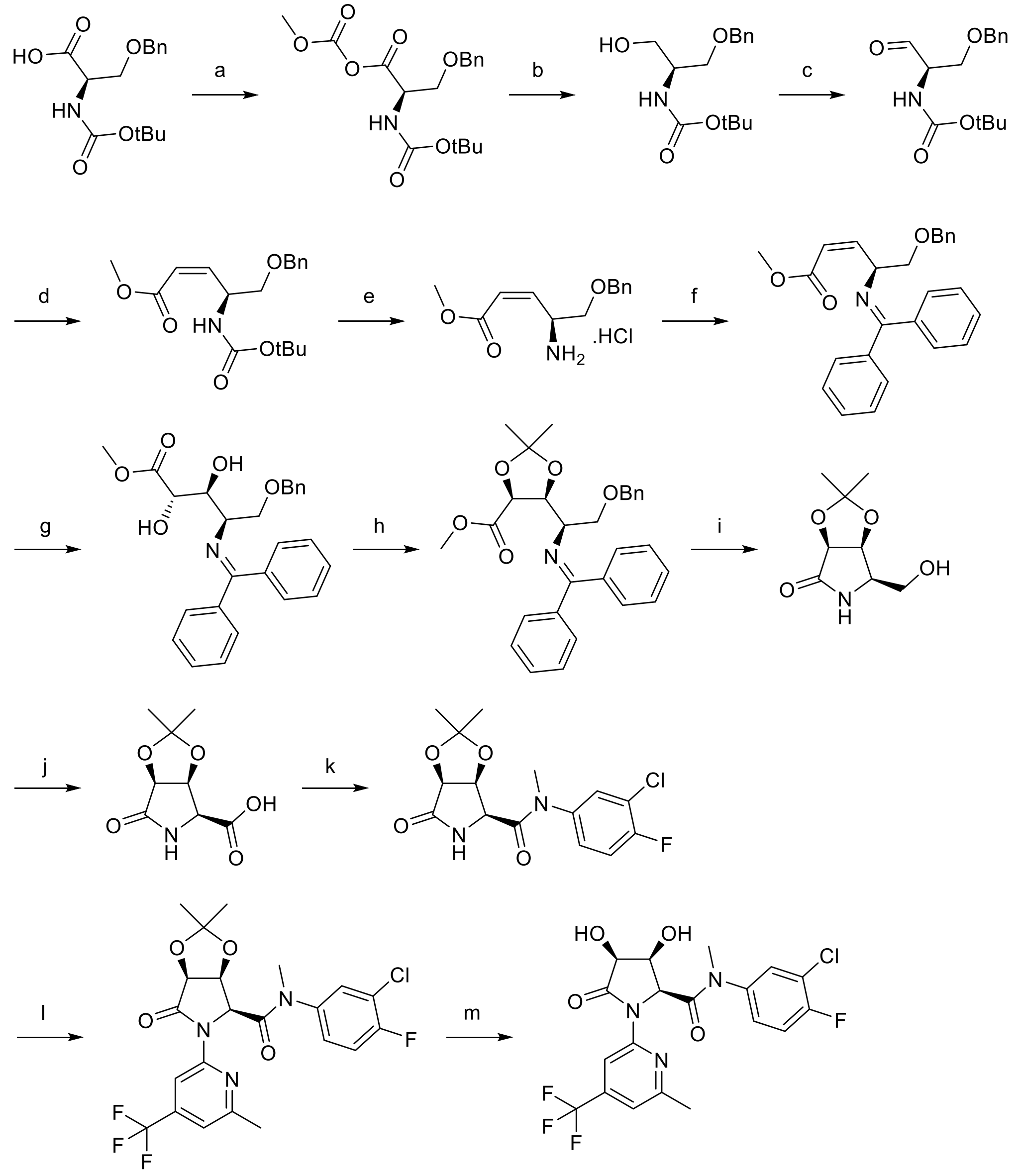

a) Methyl chloroformate, N-methylmorpholine, THF, -10°C, 30 min; b) $NaBH_4$, water, THF, rt, 15 h; c) oxalyl chloride, DCM, DMSO, -78°C, 30 min, then DIPEA, -78 to -40°C, 4 h; d) methyl 2-[bis(2,2,2-trifluoroethoxy)phosphoryl]acetate, 1M KHMDS, THF, 18-crown-6, -78°C, 2 h; e) acetyl chloride, MeOH, rt, 12 h; f) diphenylmethanimine, DCM, rt, 12 h; g) $OsO_4$, N-methylmorpholine-N-oxide, THF, 35°C, 48 h; h) pyridinium p-toluenesulfonate, 2,2-dimethoxypropane, toluene, 100°C, 12 h; i) 20% $Pd(OH)_2$/C, 10% Pd/C, MeOH, $H_2$ (20 atm), 40°C, 4 days; j) sodium periodate, $RuCl_3$, $CCl_4$, MeCN, water, 20-35°C, 3 h; k) 3-chloro-4-fluoro-N-methyl-aniline, T3P, DMF, pyridine, rt, 12 h, then 60°C, 3 h; l) 2-

bromo-6-methyl-4-(trifluoromethyl)pyridine, XantPhos, $Pd_2(dba)_3$, $Cs_2CO_3$, 1,4-dioxane, 120°C, 2 h; m) 4 M HCl, 1,4-dioxane, rt, 6 h, then 60°C, 2 h.

**Step a.** Into a 20L 4-necked round-bottom flask under inert atmosphere of nitrogen was added (2*R*)-3-(benzyloxy)-2-[(tert-butoxycarbonyl)amino]propanoic acid (CAS Number 47173-80-8; 800 g, 2.68 mol), N-methylmorpholine (298 g, 2.95 mol) and THF (8 L). The mixture was cooled to -20°C and methyl chloroformate (266 g, 2.82 mol) was added dropwise. The resulting mixture was stirred for 0.5 h at -10°C in a water/ice bath. The solids were collected by filtration to provide (2*R*)-3-(benzyloxy)-2-[(tert-butoxycarbonyl)amino]-1-[(methoxycarbonyl)oxy]propan-1-one, which was used in the next step without further purification.

**Step b.** Into a 20L 4-necked round-bottom flask under inert atmosphere of nitrogen was added water (8 L), which was cooled to 0°C before addition of $NaBH_4$ (254 g, 6.70 mol). A solution of (2*R*)-3-(benzyloxy)-2-[(tert-butoxycarbonyl)amino]-1-[(methoxycarbonyl)oxy]-propan-1-one (957 g, 2.68 mol) in THF (8 L) was added dropwise at 0°C. The resulting solution was stirred overnight at rt. The solids were removed by filtration. The filtrate was extracted with DCM (3 x 3 L) and the organic phase was concentrated. The crude was purified by silica gel chromatography (30% EtOAc in PE) to provide tert-butyl N-[(2*S*)-1-(benzyloxy)-3-hydroxypropan-2-yl]carbamate (670 g, 88% yield) as a white solid.

m/z $ES^+$ $[M+H]^+$ 282.2

**Step c.** Into a 50L 4-necked round-bottom flask under inert atmosphere of nitrogen was added a solution of $(COCl)_2$ (545 g, 4.29 mol) in DCM (15 L). This was followed by the addition of DMSO (670 g, 8.59 mol) dropwise with stirring at -78°C. After 0.5 h, the mixture was treated with a solution of tert-butyl N-[(2*S*)-1-(benzyloxy)-3-hydroxypropan-2-yl]carbamate (610 g, 2.17 mol) in DCM (3 L) dropwise with stirring at -78°C and stirred for another 0.5 h. To the mixture was added N,N-diisopropylethylamine (1664 g, 12.88 mol) dropwise with stirring at -78°C. The resulting mixture was stirred for 2 h at -78°C followed by an additional 2 h at -40°C. The reaction mixture was cooled to -70°C and

quenched by the addition to 5% aqueous $KHSO_4$ (18 L). The resulting solution was extracted with DCM (5 L). The organic phase was washed with brine, dried over $Na_2SO_4$ and concentrated. The crude was used in the next step without further purification. Note, the product was not stable in LCMS, the crude was detected by thin layer chromatography and confirmed with $^1$H NMR.

**Step d.** Into a 20L 4-necked round-bottom flask under an inert atmosphere of nitrogen was added a solution of methyl 2-[bis(2,2,2-trifluoroethoxy)phosphoryl]acetate (682 g, 2.14 mol) in THF (6 L), to which 18-crown-6 (567 g, 2.14 mol) was added. This was followed by the addition of KHMDS (1M in THF, 2.14 L, 2.14 mol) dropwise with stirring at -78°C. To this was added a solution of tert-butyl N-[(2*R*)-1-(benzyloxy)-3-oxopropan-2-yl]carbamate (605 g, 2.14 mol) in THF (1.8 L) at -78°C. The resulting reaction mixture was stirred for 2 h at -78°C. The reaction was quenched by addition of 1 M HCl (12 L). The resulting solution was extracted with EtOAc (2 x 5 L), washed with water, dried over $Na_2SO_4$ and concentrated. The crude was purified by silica gel chromatography (30% EtOAc in PE) to provide methyl (2Z,4*S*)-5-(benzyloxy)-4-[(tert-butoxycarbonyl)amino]pent-2-enoate (655 g, 90% yield) as a white solid.

m/z $ES^+$ $[M+H]^+$ 336.1; $^1$H NMR (300 MHz, DMSO-d6) δ ppm 7.36 – 7.26 (m, 5H), 6.18 – 6.12 (m, 1H), 5.90 – 5.86 (m, 1H), 5.39 – 5.32 (m, 1H), 4.55 – 4.43 (m, 2H), 3.64 (s, 3H), 3.50 – 3.39 (m, 2H), 1.47 (s, 9H).

**Step e.** Into a 5L 3-necked round-bottom flask under inert atmosphere of nitrogen was added methyl (2Z,4*S*)-5-(benzyloxy)-4-[(tert-butoxycarbonyl)amino]pent-2-enoate (655 g, 1.93 mol) and MeOH (3275 mL), followed by acetyl chloride (455 g, 5.80 mol) dropwise with stirring at 0°C. The resulting mixture was stirred for 12 h at rt. The resulting mixture was concentrated, the residue re-dissolved in THF and concentrated again. The crude was treated with n-hexane and solids were collected by filtration to provide methyl (2Z,4*S*)-4-amino-5-(benzyloxy)pent-2-enoate hydrochloride (480 g, 90% yield) as a light brown solid.

m/z $ES^+$ $[M+H]^+$ 236.1; $^1H$ NMR (300 MHz, DMSO-d6) δ ppm 8.58 (bs, 3H), 7.38 – 7.29 (m, 5H), 6.38 – 6.32 (m, 1H), 6.16 – 6.12 (m, 1H), 5.39 – 5.32 (m, 1H), 4.61 – 4.49 (m, 2H), 3.68 (s, 3H), 3.44 (bs, 2H).

**Step f.** Into a 5L 3-necked round-bottom flask under inert atmosphere of nitrogen was added DCM (2.40 L), methyl (2Z,4*S*)-4-amino-5-(benzyloxy)pent-2-enoate hydrochloride (480 g, 1.75 mol), followed by diphenylmethanimine (317 g, 1.75 mol) dropwise. The resulting mixture was stirred for 12 h at rt. The reaction mixture was concentrated to provide methyl (2Z,4*S*)-5-(benzyloxy)-4-[(diphenylmethylidene)amino]pent-2-enoate (780 g, crude) as light brown oil.

m/z $ES^+$ $[M+H]^+$ 400.2

**Step g.** Into a 20L 3-necked round-bottom flask under inert atmosphere of nitrogen was added methyl (2Z,4*S*)-5-(benzyloxy)-4-[(diphenylmethylidene)amino]pent-2-enoate (95.0 g, 1.85 mol), THF (7.80 L), water (7.80 L), N-methylmorpholine-N-oxide (543 g, 4.64 mol), followed by $OsO_4$ (23.6 g, 92.7 mmol) in 4 portions. The resulting mixture was stirred for 48 h at 35°C. The reaction mixture was cooled to rt. The resulting solution was extracted with EtOAc (2 x 5 L). The organic phase was washed with water (2 x 3 L). The mixture was dried over $Na_2SO_4$ and concentrated. The solid product was stirred in hexane and the solids were collected by filtration to provide a crude mixture of methyl (2*S*,3*S*,4*R*)-5-(benzyloxy)-4-[(diphenylmethylidene)amino]-2,3-dihydroxypentanoate and methyl (2*R*,3*R*,4*R*)-5-(benzyloxy)-4-((diphenylmethylene)amino)-2,3-dihydroxypentanoate (650 g) as a light brown solid.

m/z $ES^+$ $[M+H]^+$ 434.1

**Step h.** Into a 10L 4-necked round-bottom flask under inert atmosphere of nitrogen was added a mixture of methyl (2*S*,3*S*,4*R*)-5-(benzyloxy)-4-[(diphenylmethylidene)amino]-2,3-dihydroxypentanoate and methyl (2*R*,3*R*,4*R*)-5-(benzyloxy)-4-((diphenylmethylene)amino)-2,3-dihydroxypentanoate (650 g, 1.42 mol), toluene (6.5 L), pyridinium p-toluenesulfonate (89.5 g, 356 mmol) and 2,2-dimethoxypropane (742 g, 7.12

mol). The resulting mixture was stirred for 12 h at 100°C. The reaction mixture was concentrated. The crude product was purified by silica gel chromatography (3% EtOAc in PE) to provide methyl (4*S*,5*S*)-5-[(1*R*)-2-(benzyloxy)-1-[(diphenylmethylidene)amino]ethyl]-2,2-dimethyl-1,3-dioxolane-4-carboxylate 445 g (65% yield, over 3 steps) as light yellow oil.

m/z $ES^+$ $[M+H]^+$ 474.2; $^1H$ NMR (300 MHz, DMSO-d6) δ ppm 7.75 – 7.16 (m, 15H), 4.65 – 4.63 (m, 1H), 4.55 – 4.50 (m, 1H), 4.31 (s, 2H), 3.74 – 3.68 (m, 1H), 3.59 – 3.57 (m, 2H), 3.26 (s, 3H), 1.54 (s, 3H), 1.33 (s, 3H).

**Step i.** Into a 10L hydrogen pressure tank reactor was added methyl (4*S*,5*S*)-5-[(1*R*)-2-(benzyloxy)-1-[(diphenylmethylidene)amino]ethyl]-2,2-dimethyl-1,3-dioxolane-4-carboxylate (445 g, 930 mmol), MeOH (4.45 L), 20% $Pd(OH)_2$/C (65 g, 93 mmol) and 10% Pd/C (99 g, 93 mmol). The resulting mixture was stirred under hydrogen atmosphere (20 atm) for 4 days at 40°C. The reaction mixture was then filtered and concentrated. The crude was stirred in hexane and then collected by filtration to provide (3a*S*,6*R*,6a*S*)-6-(hydroxymethyl)-2,2-dimethyl-tetrahydro-[1,3]dioxolo[4,5-c]pyrrol-4-one (153 g, 87% yield) as a white solid.

m/z $ES^+$ $[M+H]^+$ 188.0; $^1H$ NMR (300 MHz, DMSO-d6) δ ppm 7.89 (bs, 1H), 4.77 – 4.75 (m, 1H), 4.68 – 4.65 (m, 1H), 4.57 – 4.55 (m, 1H), 3.65 – 3.55 (m, 2H), 3.47 – 3.39 (m, 1H), 1.30 (s, 6H).

**Step j.** Into a 10L 3-necked round-bottom flask under inert atmosphere of nitrogen, was added (3a*S*,6*R*,6a*S*)-6-(hydroxymethyl)-2,2-dimethyl-tetrahydro-[1,3]dioxolo[4,5-c]pyrrol-4-one (153 g, 809 mmol), MeCN (1.38 L), carbon tetrachloride (1.38 L), water (2.00 L), sodium periodate (519 g, 2.43 mol) and $RuCl_3$ (16.8 g, 80.9 mmol). The reaction mixture was stirred for 3 h at 20-35°C. The reaction mixture was then filtered and concentrated. The crude was dissolved in MeOH, filtered and concentrated to provide (3a*S*,4*S*,6a*S*)-2,2-dimethyl-6-oxo-tetrahydro-[1,3]dioxolo[4,5-c]pyrrole-4-carboxylic acid (170 g, 72%) as a light brown solid.

m/z $ES^+$ $[M+H]^+$ 202.2; $^1H$ NMR (300 MHz, DMSO-d6) δ ppm 12.86 (s, 1H), 8.19 (s, 1H), 4.88 (t, J = 5.6 Hz, 1H), 4.56 (d, J = 5.9 Hz, 1H), 4.31 (d, J = 5.3 Hz, 1H), 1.27 (d, J = 5.4 Hz,6H); $[\alpha]_D$= 13.2 degrees (C=0.22 g/100 mL in MeOH, T=21.2)

**Step k.** A mixture of (3a*S*,4*S*,6a*S*)-2,2-dimethyl-6-oxo-tetrahydro-[1,3]dioxolo[4,5-c]pyrrole-4-carboxylic acid (100 mg, 0.45 mmol), 3-chloro-4-fluoro-N-methyl-aniline (CAS Number 77898-24-9; 159 mg, 0.99 mmol), T3P (3.16 g, 4.97 mmol, 50% wt.% in DMF) in pyridine (5 mL) was degassed and purged 3 times with nitrogen, and then stirred at rt for 12 h. The reaction mixture was stirred at 60°C for another 3 h. The reaction mixture was evaporated and the crude residue purified by reverse phase column chromatography to afford (3a*S*,4*S*,6a*S*)-N-(3-chloro-4-fluorophenyl)-N,2,2-trimethyl-6-oxotetrahydro-3aH-[1,3]dioxolo[4,5-c]pyrrole-4-carboxamide (100 mg, 59% yield) as a yellow solid.

$^1H$ NMR (400 MHz, $CD_3OD$) δ ppm 7.65 (d, J = 5.2 Hz, 1H), 7.43-7.38 (m, 2H), 4.48 (d, J = 6.0 Hz, 1H), 4.41 (d, J = 5.2 Hz, 1H), 4.06-4.04 (m, 1H), 3.66 (s, 3H), 1.40 (s, 3H), 1.32 (s, 3H).

**Step l.** A mixture of (3a*S*,4*S*,6a*S*)-N-(3-chloro-4-fluorophenyl)-N,2,2-trimethyl-6-oxo-tetrahydro-3aH-[1,3]dioxolo[4,5-c]pyrrole-4-carboxamide (80 mg, 0.23 mmol), 2-bromo-6-methyl-4-(trifluoromethyl)pyridine (84 mg, 0.35 mmol), XantPhos (27 mg, 0.047 mmol), $Pd_2(dba)_3$ (21 mg, 0.023 mmol) and Cs*2*CO*3* (152 mg, 0.47 mmol) in 1,4-dioxane (4 mL) was degassed and purged 3 times with nitrogen and then stirred at 120°C for 2 h. The reaction mixture was evaporated and the crude residue purified by column chromatography (20% EtOAc in PE) to afford (3a*S*,4*S*,6a*S*)-N-(3-chloro-4-fluorophenyl)-N,2,2-trimethyl-5-(6-methyl-4-(trifluoromethyl)pyridin-2-yl)-6-oxotetrahydro-3aH-[1,3]dioxolo[4,5-c]pyrrole-4-carboxamide (60 mg, 46% yield) as a yellow oil.

$^1H$ NMR (400 MHz, $CD_3OD$) δ ppm 8.47 (s, 1H), 7.77 (d, J = 6.4 Hz, 1H), 7.60-7.58 (m, 1H), 7.52-7.47 (m, 1H), 7.32 (s, 1H), 5.20 (d, J = 6.8 Hz, 1H), 4.80 (d, J = 6.8 Hz, 1H), 4.54 (d, J = 6.8 Hz, 1H), 3.25 (s, 3H), 2.64 (s, 3H), 1.47 (s, 3H), 1.41 (s, 3H).

**Step m.** A solution of (3a*S*,4*S*,6a*S*)-N-(3-chloro-4-fluorophenyl)-N,2,2-trimethyl-5-(6-methyl-4-(trifluoromethyl)pyridin-2-yl)-6-oxotetrahydro-3aH-[1,3]dioxolo[4,5-c]pyrrole-4-carboxamide (60 mg, 0.1 mmol) in HCl in 1,4-dioxane (4 M, 2 mL) was stirred at rt for 6 h, then at 60°C for 2 h. The reaction mixture was evaporated and the crude residue purified by reverse phase column to afford the title compound (32 mg, 56% yield) as yellow solid.

m/z ES$^+$ $[M+H]^+$ 462.0; $^1$H NMR (400 MHz, $CD_3OD$) δ ppm 8.41 (s, 1H), 7.83 (dd, J = 6.4 Hz, 2.0 Hz, 1H), 7.64-7.61 (m, 1H), 7.49-7.44 (m, 1H), 7.29 (s, 1H), 5.12 (d, J = 5.2 Hz, 1H), 4.26-4.19 (m, 2H), 3.27 (s, 3H), 2.64 (s, 3H).

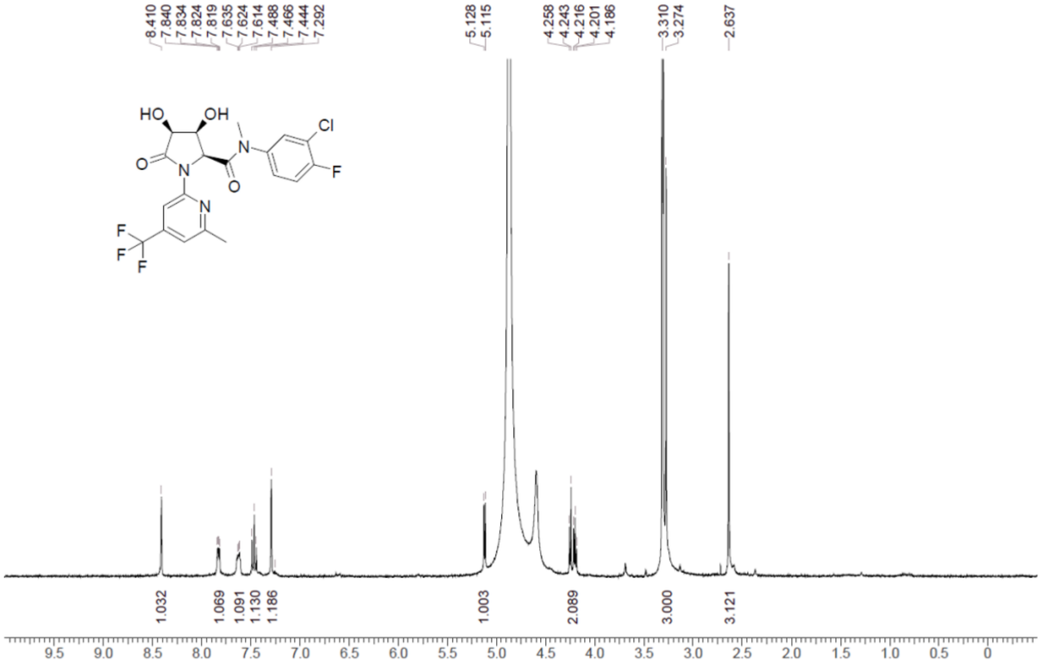

**ART558 Kinase selectivity assay.** Kinase selectivity was assessed against a panel of enzymes at Eurofins. (https://www.eurofinsdiscoveryservices.com/services/in-vitro-assays/kinases/screening-profiling-services/)

| | **ART558 @ 10 μM** |
|---|---|
| Abl(h) | 92 |
| Abl (M351T)(h) | 86 |
| Arg(h) | 107 |
| Aurora-A(h) | 84 |
| Bmx(h) | 99 |
| BTK(h) | 95 |
| CaMKIIβ(h) | 110 |
| CaMKIV(h) | 89 |
| CDK1/cyclinB(h) | 106 |
| CDK2/cyclinA(h) | 117 |
| CDK2/cyclinE(h) | 101 |
| CDK3/cyclinE(h) | 90 |
| CDK5/p35(h) | 93 |
| CDK6/cyclinD3(h) | 96 |
| CDK7/cyclinH/MAT1(h) | 97 |
| CHK1(h) | 74 |
| CHK2(h) | 106 |
| CK1δ(h) | 94 |
| CK1(y) | 104 |
| CK2(h) | 101 |
| CSK(h) | 115 |
| c-RAF(h) | 89 |
| cSRC(h) | 101 |
| EGFR(h) | 104 |
| EphB2(h) | 95 |
| EphB4(h) | 96 |
| FGFR3(h) | 99 |
| Flt3(h) | 109 |
| Fms(h) | 104 |
| Fyn(h) | 87 |
| GSK3β(h) | 98 |
| IGF-1R(h) | 97 |
| MAPK1(h) | 104 |
| MAPK2(h) | 105 |
| MEK1(h) | 95 |
| Met(h) | 102 |
| MST2(h) | 100 |
| NEK2(h) | 95 |
| p70S6K(h) | 95 |
| PAK2(h) | 104 |
| PAR-1Bα(h) | 103 |
| PDGFRα(h) | 106 |
| PDGFRβ(h) | 102 |
| PDK1(h) | 100 |
| PKA(h) | 82 |

| | |
|---|---|
| PKBα(h) | 104 |
| PKBβ(h) | 83 |
| PKBγ(h) | 98 |
| PKCα(h) | 99 |
| PKCβII(h) | 101 |
| PKCγ(h) | 112 |
| PKCδ(h) | 101 |
| PKCε(h) | 108 |
| PKCη(h) | 101 |
| PKCι(h) | 84 |
| PKCμ(h) | 95 |
| PKCθ(h) | 92 |
| PKCζ(h) | 90 |
| PKD2(h) | 116 |
| PRAK(h) | 85 |
| PRK2(h) | 108 |
| Ros(h) | 122 |
| Rsk1(h) | 93 |
| Rsk1(r) | 97 |
| Rsk2(h) | 101 |
| Rsk3(h) | 108 |
| SGK(h) | 88 |
| Syk(h) | 100 |
| Tie2 (h) | 82 |
| Wee1(h) | 100 |
| Yes(h) | 92 |
| ATM(h) | 118 |
| ATR/ATRIP(h) | 92 |
| DNA-PK(h) | 93 |
| PI3 Kinase (p110α(E545K)/p85 α)(m) | 91 |
| PI3 Kinase (p110 α (H1047R)/p85 α)(m) | 98 |
| PI3 Kinase (p110 α (E542K)/p85 α)(m) | 89 |
| PI3 Kinase (p110 α /p85 α)(h) | 113 |

**Recombinant Protein Expression.** Recombinant human full length Polθ (fl- Polθ) containing an N-terminal $His_6$ tag and a C-terminal FLAG tag (His Polθ- [FL]-FLAG) was produced using the Bac-to-Bac Baculovirus expression system (Thermo Fisher Scientific) using protocols modified from (8, 47). DNA was synthesised at GeneArt (Thermo Fisher Scientific) and cloned into pFastBacHTc incorporating a FLAG tag at the C-terminus. DH10Bac cells were transformed with pFastBacHTc-Polq (FL)-FLAG and incubated with shaking (120 rpm) at 37 $^{o}$C for 6 hours. Serial dilutions of the transformed cells ($10^{-1}$, $10^{-2}$ and $10^{-3}$) with S.O.C. medium were plated out onto LB agar plates containing 50 μg/mL kanamycin, 7 μg/mL gentamycin, 10 μg/mL tetracycline, 100 μg/mL Bluo-gal and 40 μg/mL IPTG, and incubated for 2 days at 37$^{o}$C. White colonies were isolated and grown overnight in 4 mL LB agar medium containing 50 μg/mL kanamycin, 7 μg/mL gentamycin and 10 μg/mL tetracycline. The His-Polq (FL)-FLAG bacmids were purified following Bac-to-Bac standard protocols (Thermo Fisher Scientific). 5 μL of purified recombinant bacmid DNA, along with Cellfectin™ II Reagent

(Thermo Fisher Scientific), were used to transfect Sf9 cells in 6 well plates. After four days post-transfection His-Polq (FL)-FLAG P1 baculoviruses were harvested and titered by plaque assay. For virus amplification, His- Polθ (FL)-FLAG recombinant baculovirus was amplified by infecting multiple 200 mL Sf9 suspension cultures (1x$10^6$ cells/mL) in 1 L Erlenmeyer flasks with P1 virus at a MOI of 0.05. The cells were incubated at 28 °C, 120 rpm for 3 days in serum free Sf-900 II media (Thermo Fisher Scientific). The virus-containing supernatant was harvested by centrifugation at 1500 rpm for 10 min at 4 $^o$C, filter sterilized and supplemented with 5% fetal calf serum (FCS). The amplified virus was titred by plaque assay, as standard. For expression of recombinant His- Polθ (FL)-FLAG protein, 5 L suspension cultures of Sf9 insect cells (1x$10^6$ cells/mL) were infected with high titre virus at a MOI of 5 and grown in 10 L Cellbags (GE Healthcare). Using a WAVE Bioreactor System 20/50EHT, the cells were incubated for 48 hours at 28 $^o$C with a rocking speed of 20 rpm, rocking angle of 7$^o$ and an aeration rate of 0.2 lpm. 48 hours post-infection, the cells were harvested by centrifugation at 1500 rpm for 10 min. The cell pellets were washed with ice-cold Dulbecco's PBS (1x) without Ca & Mg (PAA Laboratories Ltd) and the cells centrifuged for further 25 min at 1500 rpm. The cell pellets were flash frozen in liquid nitrogen and stored at -80 $^o$C.

The purification of full-length Polθ was carried out at 4 $^o$C and completed within 1 day. Pellets from a 5 L culture were resuspended in 300 mL lysis buffer (100 mM Tris-HCl pH 8, 0.6 M ammonium sulphate, 10 % (v/v) glycerol, 0.5 % (v/v) NP-40, 10 mM EDTA, 1 mM AEBSF, Protease inhibitor cocktail VIII (Fisher), Complete protease inhibitors (Roche), 5 µL of Base Muncher (Expedeon)). Lysis was carried out by gentle sonication on ice (Qsonica Q700, 30% amplitude, ½" probe, 3 x 10s ON/50s OFF). An additional 5 µL Base Muncher was then added. The lysate was centrifuged at 49000 g for 30 mins, and the supernatant was incubated with rolling for 1 hr at 4 °C with 5 mL of FLAG resin (Sigma-Aldrich), equilibrated in Flag wash buffer (50 mM Tris-HCl pH 8, 1 M NaCl, 10 % (v/v) glycerol, 0.1 % (v/v) NP-40 and 1 mM EDTA). The beads were split between two empty column cases and washed with 10 column volumes (CV) of Flag wash buffer, and then with 2 CV Flag wash buffer (EDTA-free). Protein was eluted with 6 mL Flag elution buffer (50 mM Tris-HCl pH 8, 1 M NaCl, 10 % (v/v) glycerol, 0.1 % (v/v) NP-40, 200 µg/mL 3x Flag peptide (Sigma-Aldrich)) using 3 mL/column. All column steps were performed by gravity flow. The eluted protein was passed over 2 mL of Ni Sepharose 6 Fast Flow resin (GE Healthcare), equilibrated in IMAC buffer (50 mM Tris-HCl pH 8, 500 mM NaCl, 10 % (v/v) glycerol, 0.1 % (v/v) NP-40, 1 mM BME and 25 mM Imidazole). The flow through was collected and reapplied to the column. The beads were washed with 5 CV of IMAC buffer. Protein was eluted in 6 mL IMAC Buffer supplemented with 250 mM Imidazole. The protein was snap frozen in liquid nitrogen and stored at -80 °C.

Human Polθ polymerase domain (aa1820-2590) (pd-Polθ) was expressed and purified from *E.coli*. The cDNA was cloned into pET21a incorporating a His-humanSUMO tag at the N terminus. pET21a-His-hSUMO3- Polθ (1820-2590) was transformed into Rosetta2 (DE3) pLysS and grown on LB agar, 100 µg/mL ampicillin, 35 µg/mL chloramphenicol and incubated at 37 °C overnight. Transformed cells were cultured in Terrific Broth media at 30 °C using 220 rpm until $OD_{600nm}$ = 0.6, at which point the temperature was reduced to 18 °C for 2 hours. The cells were induced with 0.2 M IPTG and further incubated at 18 °C

and 220 rpm for 16 hours. Cells were harvested by centrifugation at 6000 g for 20 minutes and pellets washed with PBS before storing at -20 °C.

The purification of pd-Polθ was carried out at 4 °C. Cell material was resuspended in 50 mM HEPES pH 8.0, 300 mM NaCl, 10% (v/v) glycerol, 1.5% (v/v) NP-40, 1 mM EDTA, 2 mM TCEP, 1 Sigmafast tablet per 100 mL, 1 U/mL Benzonase (Merck)) at 10% (w/v) before passing through a constant systems cell disruptor (30K psi). The lysate was centrifuged at 40,000 g for 1 hour and the supernatant then passed through a 0.22 µm vacuum filter. The sample was applied to a HisTrap Excel column (GE Healthcare) equilibrated in IMAC buffer (50 mM HEPES pH 8.0, 300 mM NaCl, 10% (v/v) glycerol, 0.0025% (v/v) NP-40, 1 mM EDTA, 1 mM TCEP). The column was washed with 10 CV of IMAC buffer containing additional 10 mM imidazole, then with 5 CV of IMAC buffer supplemented with 1M NaCl, followed by 10 CV of IMAC buffer supplemented with 20 mM imidazole. The protein was eluted with 50 mM HEPES pH 8.0, 300 mM NaCl, 250 mM imidazole, 10% (v/v) glycerol, 0.0025% (v/v) NP-40, 1 mM EDTA, 1 mM TCEP.

Eluted material was further purified on a HiTrap Heparin column (GE Healthcare) equilibrated in Heparin buffer (50 mM HEPES pH 8.0, 300 mM NaCl, 10% (v/v) glycerol, 0.0025% (v/v) NP-40, 1 mM EDTA, 1 mM TCEP). pd-Polθ was eluted with a 20 CV gradient of 0.3-1 M NaCl in Heparin buffer. pd-Polθ was then buffer exchanged into 50 mM HEPES pH 8.0, 200 mM NaCl, 10% (v/v) glycerol, 0.0025% (v/v) NP-40, 1 mM EDTA, 1 mM TCEP using ZebaSpin columns (Thermo Fisher Scientific). SUMO protease 2 (Life Sensors) was added to the sample at 1 U protease per 50 µg protein and incubated at 4 °C for 2 hours, after which the sample was filtered through a 0.22 µm syringe filter. The cleaved material was removed by passing over a Histrap Excel column (GE Healthcare). The flow through and wash steps were pooled and concentrated in a 30 KDa MWCO centrifugal concentrator (Millipore). For the final step a Superdex200 column (GE Healthcare) was equilibrated with 40 mM Tris-HCl pH 7.5, 150 mM ammonium acetate, 150 mM KCl, 1% (v/v) glycerol, 2.5 mM TCEP and the sample was applied and run over 1.2 CV. pd- Polθ was pooled and concentrated to 4-5 mg/mL using a 30 KDa MWCO centrifugal concentrator (Millipore). The pure material was concentrated, snap frozen in liquid nitrogen and stored at -80°C.

**Biochemical inhibition of Polθ helicase activity.** The biochemical activity of ART4215 on the ATPase activity of Polθ was determined by measuring the inhibition of human full-length Polq and helicase-domain Polθ in an ADP Glo activity assay. ART558 did not inhibit the helicase domain ATPase activity of either full-length or helicase-domain Polθ at the concentration range tested ($IC_{50}$ >12 mM).

**Polθ helicase protein production method.** Recombinant Polθ-HD containing an N-terminal His6tag and TEV protease cleavage site (His-TEV- Polθ-HD) was produced using the Bac-to-Bac Baculovirus expression system (Thermo Fisher Scientific). DNA was synthesised at ATG Biosynthetics and cloned into the baculovirus transfer vector pFB-LIC-Bse and transformed into E. coli DH10Bac, plated onto LB-agar containing antibiotics (50 µg/mL kanamycin, 7 µg/mL gentamycin, 10 µg/mL tetracycline) and blue-white selection agents (100 µg/mL Bluo-gal, 40 µg/mL IPTG) and incubated for 24-48hrs at 37°C. White colonies were re-streaked onto fresh plates and incubated for another 24 to 48 h at 37°C. White colonies were isolated and grown overnight in LB medium

containing antibiotics (50 µg/mL kanamycin, 7 µg/mL gentamycin, 10 µg/mL tetracycline) at 37 °C. The His-TEV-Polθ-HD bacmids were purified following Bac-to-Bac standard protocols (Thermo Fisher Scientific). 5 µL of purified recombinant bacmid DNA, along with Cellfectin™ II Reagent (Thermo Fisher Scientific), were used to transfect Sf21 cells in 6 well plates and incubated at 27 °C for 72-96 h. His-TEV-Polθ-HD P1 baculoviruses were harvested and titred by plaque assay. For virus amplification, His-TEV-Polθ-HD recombinant baculovirus was amplified by infecting 50 mL Sf21 suspension cultures (1x106 cells/mL) with P1 virus at a MOI of 0.05. The cells were incubated at 27 °C, 120 rpm for 3 days in serum free Sf-900 II media (Thermo Fisher Scientific). The virus-containing supernatant was harvested by centrifugation at 2000 g for 20 min at 4 °C, then stored at 4 °C. The amplified virus was titred by plaque assay, as standard.

For expression of recombinant His-TEV-Polθ-HD protein, 2.5 L suspension cultures of Sf21 insect cells (0.65x106 cells/mL) were grown in 2.5 L SF900-II SFM media containing 5 µg/mL gentamycin in 5 L Optimum Growth shake flasks at 0.65x106 cells/mL and allowed to double in density overnight at 27 °C on orbital shakers at 110 rpm. The overnight cultures were counted and then infected with high titre virus at a MOI of 5. The infected cultures were incubated for 48 h at 110 rpm, 27 °C. Cells were harvested by centrifugation at 3,400 g for 15 min at 4 °C. The cell pellets were flash frozen in liquid nitrogen and stored at -80 °C.

The purification of His-TEV-Polθ-HD was carried out at 4 °C. Cell material was resuspended in 50 mM HEPES pH 7.5, 500 mM NaCl, 5 % glycerol, 10 mM imidazole, 0.5 mM TCEP, Benzonase 10 U/ mL, Complete Inhibitor tablets w/o EDTA from Roche (2 per 100 mL) and 10 U/mL Benzonase at 20% (w/v) before passing through a cell disruptor at 25 K psi. Lysate was centrifuged at 200,000 g for 30 minutes. Supernatant was applied to a HisTrap FF crude column (Cytiva) equilibrated in Wash buffer (50 mM HEPES pH 7.5, 500 mM NaCl, 5 % glycerol, 30 mM imidazole, 0.5 mM TCEP), and washed with 10 column volumes (CV) of the same buffer before eluting with a 20 CV gradient to 100% Elution buffer (50 mM HEPES pH7.5, 500 mM NaCl, 5 % glycerol, 500 mM imidazole, 0.5 mM TCEP). The pooled elution sample was incubated with AcTEV protease (Thermo Fisher Scientific) overnight at 4 °C in dialysis against SEC buffer (20 mM HEPES pH 7.5, 500 mM NaCl, 5% Glycerol, 0.5 mM TCEP). The sample was passed back over the HisTrap FF column (Cytiva) and washed with 10 CV of Wash buffer. Flow-through and wash steps were pooled and concentrated to 30 mL in a 50 KDa MWCO Amicon centrifugal concentrator (Merck). SEC was then carried out over two runs on a 26/60 S200 column (Cytiva) in SEC buffer. The main elution peak was pooled and concentrated to 1.3 mg/mL in a 50 KDa MWCO Amicon centrifugal concentrator (Merck).

***Polθ* Biochemical ADP Glo Assay.** Compounds were dispensed into white 384-well Proxiplates (PerkinElmer) using an Echo 550 (Labcyte). For each compound, 40 nL of a 2-fold dilution series was dispensed in duplicate starting from a 1.2 mM top concentration of compound in DMSO. This gave a final in-assay top concentration of 12 mM and 1% (v/v) DMSO concentration.

Reactions were performed at room temperature, in freshly prepared assay buffer (25 mM Tris-HCl pH 7.5, 6 mM NaCl, 1.5 mM MgCl2, 5% (v/v) glycerol, 0.01% v/v Triton x-100, 0.01% (w/v) Bovine γ-Globulin, 1 mM dithiothreitol). All additions were made using either electronic multichannel pipettes (Integra) or the Tempest liquid handler (Formulatrix). Single stranded DNA was purchased from Sigma (30mer ssDNA (CT)15)

2 mL of 2x helicase domain Polθ and DNA mix (2 nM helicase domain Polq and 2 nM DNA in assay buffer) was added to plates that had been pre-dispensed with compound except for the blank wells to which was added 2 µL 2x DNA in assay buffer. The plates were covered and left to incubate for 30 minutes at room temperature before starting the enzyme reaction with the addition of 2 mL of 2x substrate mix (80 mM ATP in assay buffer). The plates were covered and left to incubate for 1 hour at room temperature before the addition of the ADP Glo detection reagents. 4 µL ADP Glo was added and plates incubated for 40 minutes before addition of 8 µL kinase detection reagent. After the addition of the kinase detection reagent, the plates were covered and incubated for 40 minutes before being read on a PHERAstar FS plate reader (BMG Labtech) using an LUM plus optic module. The gain was set to 3600, focal height 12.8 mm.

$IC_{50}$ data were analysed in Abase (IDBS). Percent inhibition was calculated as follows: 100-((Compound-Min) / (Max-Min) *100) where "Max" is the high control (DMSO) and "Min" is the no enzyme control. IC50 values were calculated using a four-parameter logistic curve fit using the following formula: LowerBound+((UpperBound-LowerBound)/(1+((IC50/x)^Hill)))

An analogous method was used for determination of helicase activity inhibition using full length protein.

**DNA complex preparation**. Oligonucleotides used are listed in Table S2.

**Mechanism of action assays.** Initial rate data were globally fitted to four models of reversible inhibition: competitive (equation (1)), uncompetitive (equation (2)), mixed (equation (3)) and noncompetitive (equation (4)), using GraphPad Prism V.8.4.2. GraphPad's inbuilt Akaike information criteria (AIC) function was employed as a statistical test to aid model selection (for example, noncompetitive *versus* mixed).

$$v_o = \frac{v_{max}[S]}{K_M\left(1 + \frac{[I]}{K_{ic}}\right) + [S]}$$

(equation 1)

$$v_o = \frac{v_{max}[S]}{K_M + [S]\left(1 + \frac{[I]}{K_{iu}}\right)}$$

(equation 2)

$$v_o = \frac{v_{max}[S]}{K_M\left(1 + \frac{[I]}{K_{ic}}\right) + [S]\left(1 + \frac{[I]}{K_{iu}}\right)}$$

(equation 3)

$$v_o = \frac{v_{max}[S]}{K_M\left(1+\frac{[I]}{K_i}\right)+[S]\left(1+\frac{[I]}{K_i}\right)}$$
(equation 4)

**siRNA transfection.** siRNAs used are listed in Supplementary Table 2.

**Viability and clonogenic survival assays.** For ART558 sensitivity, exponentially growing cells were seeded in 6-well or 24-well plates at a concentration of 1000–2000 cells per well. Cells were continuously exposed to the compound with media and drug replaced every 72 h. After 12 or 14 days, cells were fixed and stained with sulphorhodamine-B (Sigma) and colonies were counted. Alternatively, at the end of the assay, cell viability was estimated using Cell-Titer Glo (Promega). Graphpad Prism software (v8.4.2) was used to convert SF into four parameter logistic regression dose/response curves.

Alternatively, cells in exponential growth phase were detached with trypsin, counted and resuspended in media at the density indicated in table below. 1mL of cells were seeded per well in a 24-well plate in triplicate and incubated overnight at 37$^{o}$C. The day after, cells were treated with a seven-point dose response curve with a one-in-three serial dilution of POLθi ART558 (top concentration for MDA-MB-436 models was 6 µM) for the timepoints listed in table below. For MDA-MB-436 cells media was replenished every five days. For DLD-1, cells were treated with a one-in-three serial dilution of olaparib (S1060, SelleckChem) (top concentration 300 nM) combined with a single concentration of ART558 at the concentrations indicated on the graphs. In all assays the DMSO vehicle final concentration was normalized across the whole plate and kept below 0.5%. RPMI1640 + FBS media was used for all the cell lines listed below.

*Colony Formation Assay Seeding Densities and Endpoints*

| Cell Line | Population | Density (cells/well) | Experimental endpoint (days) |
|---|---|---|---|
| MDA-MB-436 | parental | 3000 | 14 |
| MDA-MB-436 | SHLD2 KO clone D1 | 6000 | 14 |
| MDA-MB-436 | SHLD2 KO clone D1 restored | 6000 | 14 |
| MDA-MB-436 | SHLD2 KO clone G1 | 6000 | 14 |
| MDA-MB-436 | SHLD2 KO clone G1 restored | 6000 | 14 |
| DLD-1 | parental | 100 | 9 |
| DLD-1 | BRCA2 KO | 450 | 13 |

At the experimental endpoint cells were fixed with 70% Ethanol for 20 minutes at room temperature with shaking. Cells were then stained with 0.04% Crystal Violet (Sigma

Aldrich) for 20 minutes at room temperature with shaking. Cells were washed 6 times with water and air-dried overnight.

Plates were imaged using a Gelcount (Oxford Optronix), and colonies were counted using parameters optimized for each cell line. DLD-1 viability curves were generated from the colony counts, while MDA-MB-436 viability curves were generated from solubilized colonies since the colony morphology did not allow a reliable count. Colonies were solubilized using 10% Acetic Acid (VWR) for 30 minutes, absorbance at 595nm was read using the Clariostar plate reader (BMG Labtech), and blank correction was applied.

**Immunoblotting.** Primary antibodies used are as follows:

| **Protein target** | **Manufacturer** | **Catalog number** | **Used for:** | **Dilution** |
|---|---|---|---|---|
| POLQ | Gift from J.S. Hoffman | doi: 10.1038/ncomms5285 | WB | 1:5000 |
| BRCA1 | Santa Cruz | Sc-6954 | WB | 1:500 |
| 53BP1 | Antibodies-online | ABIN1724821 | WB | 1:1000 |
| TP53BP1 | Bethyl | A300-272A | WB | 1:1000 |
| SHLD2 | Generated for Artios Pharma by CRB | N/A | WB | 1:500 |
| EXO1 | Bethyl | A302-640A | WB | 1:500 |
| GAPDH | Cell signalling technologies | 3683S | WB | 1:5000 |
| Vinculin | Santa Cruz | sc-73614 | WB | 1:5000 |
| Tubulin | Abcam | Ab7291 | WB | 1:5000 |
| β-Actin | Cell signalling technologies | 13E5 | WB | 1:5000 |
| γH2Ax | MerckMillipore | 05-636 | WB + IF | 1:2000 |
| BrdU | GE Healthcare | RPN202 | IF | 1:500 |
| RPA | Abcam | ab2175 | IF | 1:1000 |
| PCNA | Santa Cruz | sc7907 | IF | 1:1000 |
| pRPA (S4/S8) | Bethyl | A300-245A | IF | 1:1000 |

Secondary antibodies used in Western blot studies (dilution 1:10000):
IRDye® 680RD Donkey anti-Mouse IgG - Li-Cor 926-68072
IRDye® 800CW Goat anti-Rabbit IgG - Li-Cor 926-32211
Goat anti-Rabbit IgG HRP - Sigma A9169-2ML
Goat anti-mouse IgG HRP - Invitrogen 31430-2ML

Secondary antibodies used in immunofluorescence studies (dilution 1:1000)
Alexa Fluor 555–conjugated mouse - Thermo Fisher Scientific A-21422
Alexa Fluor 488–conjugated rabbit - Thermo Fisher Scientific A-11034

***In vivo* PK studies - related to Supplementary Figure 2f.** Microsomal stability and plasma protein binding assays were performed at WuXi Apptec using standard protocols

(https://labtesting.wuxiapptec.com/dmpk-services/in-vitro-adme/). Fasted male SD rats were used for in vivo PK studies. The test article was dissolved at 1.5 mg/mL in 10% DMSO, 10% Solutol HS15, 80% water for IV dosing; 5.0 mg/mL in 10% DMSO, 10% Solutol HS15, 80% water for the oral low dose and at 50 mg/mL in 50% Labrasol (50% water) for the 200 mg/kg oral dose. Plasma concentration versus time data was analyzed by non-compartmental approaches to derive PK parameters using the Phoenix WinNonlin 6.3 software.

## Supplementary Figures

**Supplementary Figure 1. Related to Figure 2**

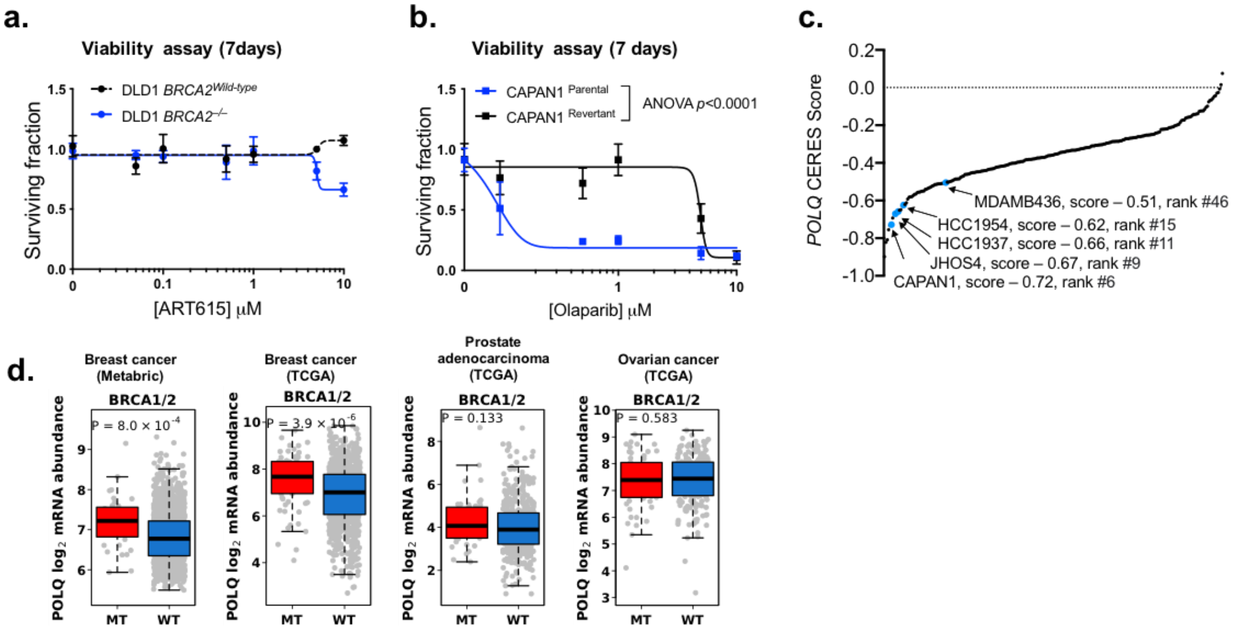


**Supplementary Figure 1. ART558 elicits *BRCA2* synthetic lethality - Related to figure 2.** (**a**) Dose-response survival curves of DLD1.*BRCA2*$^{wild\ type}$ and DLD1.*BRCA2*$^{-/-}$ cells exposed to ART615 for five days. Cell viability was estimated by CellTiter-Glo. Data are mean surviving fractions ± SD, *n*=3. (**b**) Dose-response Olaparib survival curves in *BRCA2* mutant CAPAN1$^{Parental}$ (*BRCA2 c.6174delT*, p.S1982fs*22) and a CAPAN1 daughter clone with a *BRCA2* reversion mutation, CAPAN1$^{Revertant}$ (*BRCA2 c.6174delT;6182del5* [23]). Cell viability was estimated by CellTiter-Glo reagent after seven days drug exposure. Data are mean Surviving Fractions ± SD, *n*=3. Two-way ANOVA was used to calculate the *p* value. **(c)** Sensitivity of 249 tumour cell lines to *POLQ* CRISPR-Cas9 targeting. POLQ CERES scores from 249 tumour cell lines described on depmap.org as part of the Sanger CRISPR screen dataset. Out of 249 tumour cell lines, CAPAN1 were the 6$^{th}$ most sensitive to POLQ CRISPR-Cas9 targeting as highlighted. Sensitivity of tumour cell lines with deleterious BRCA1 mutations is also shown (MDAMB436, HCC1954, HCC1937, JHOS4). Data downloaded from depmap.org 10$^{th}$ Feb. 2021. **(d)** *POLQ* mRNA expression *vs BRCA1/2* gene status from four datasets: Breast Cancer (Metabric), Breast Cancer (TCGA), Prostate adenocarcinoma (TCGA) and Ovarian cancer (TCGA). Wilcoxon rank sum test (two-sided) was used to assess difference in POLQ mRNA abundance of BRCA1/2 MT and WT groups. Boxplots show 1st quartile (Q1), median and 3rd quartile (Q3), with whiskers plotted at the points closest to Q1 – 1.5 × IQR and Q3 + 1.5 × IQR.

**Supplementary Figure 2. Related to Figure 3**

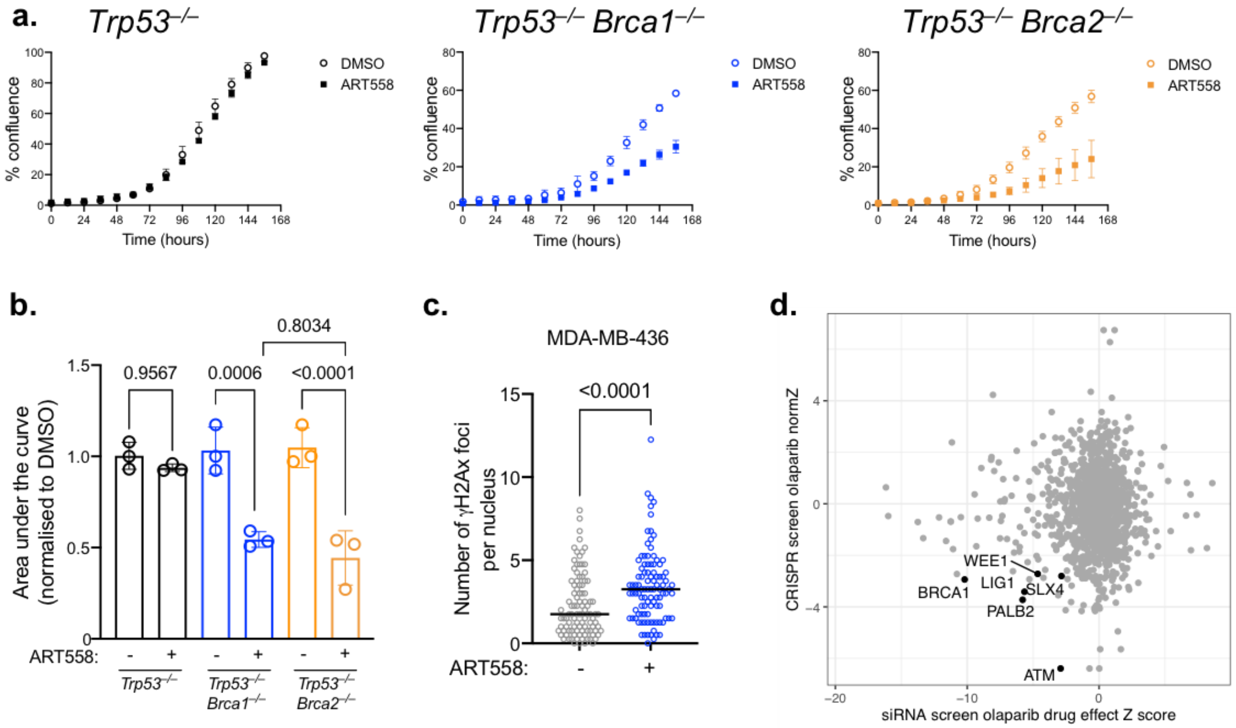


**Supplementary Figure 2. ART558 elicits *BRCA1* synthetic lethality - Related to Figure 3. (a)** Incucyte generated confluence from ID8 *Tp53*$^{mut}$ , *Tp53*$^{mut}$ *Brca1*$^{mut}$ and *Tp53*$^{mut}$ *Brca2*$^{mut}$ cells exposed to ART558 for six days. Data are mean ± SD, *n*=3 independent experiments **(b)** Bar chart illustrating the surviving fraction for each cell line, normalised to its DMSO control. Data are mean ± SD, *n*=3. One-way ANOVA with Tukey post hoc test was used to calculate *p* values. **(c)** Dot plot illustrating number of γH2AX foci per nucleus in MDA-MB-436 tumour cells following ART558 exposure. Data are means ± SD, n=100. One-way ANOVA with Tukey post hoc test was used to calculate *p* values. **(d)** Comparison of CRISPR and siRNA screen for olaparib sensitivity in RPE1 cells. NormZ values from screen data from Zimmerman *et al.* is plotted against drug effect Z score from the screen carried out in this study. Key DDR genes scoring as olaparib sensitisers in both screens are highlighted

**Supplementary Figure 3 (next page). Defects in the Shieldin complex cause PARP inhibitor resistance but Polθ inhibitor synthetic lethality.** (**a**) *SHLD2* copy number *vs. SHLD2* mRNA expression plots indicate that reduced *SHLD2* mRNA levels are associated with *SHDL2* copy number loss. Kruskal-Wallis test was used to assess the relationship between SHLD2 mRNA abundance and copy number status. Boxplots show 1st quartile (Q1), median and 3rd quartile (Q3), with whiskers plotted at the points closest to Q1 – 1.5 × IQR and Q3 + 1.5 × IQR. (**b**) *SHLD2* mRNA expression *vs. PTEN* copy number plots indicate that reduced *SHLD2* mRNA levels are associated with *PTEN* copy number loss. Kruskal-Wallis test was used to assess the relationship between SHLD2 mRNA abundance and PTEN copy number status. Boxplots show 1st quartile (Q1), median and 3rd quartile (Q3), with whiskers plotted at the points closest to Q1 – 1.5 × IQR and Q3 + 1.5 × IQR. (**c**) Western blot showing SHLD2 expression in MDA-MB-436 *SHLD2* knockout clones G1 and D1 and in SHLD2 cDNA complemented derivates (SHLD2 rest). Tubulin was used as a loading control. (**d**) Sequences of mutant *SHLD2* alleles in clones G1 and D1. (**e**) Structure and biochemical $IC_{50}$ activity of the DNA polymerase Polθ inhibitors ART812 and ART490. (**f**) Summary table of PK parameters. (**g**) Dose response survival curve for MDA-MB-436 cells (parental), and SHLD2 defective subclone (KO), clone D1. Cells were exposed to ART812 in a colony formation assay as in Figure 3i. Data are mean Surviving Fractions ± SD, n=3. Experiment representative of a biological duplicate. (**h**) Relative body weight changes in rats following treatment with 100 mg/kg ART812 and vehicle over the 76 days treatment period. Data are Mean ±SEM, for each group *n=7.* **(I,j)** Dose-response survival curve for SUM149 daughter clones with either *53BP1*, *SHLD1* or *SHLD3* defects exposed to ART558 (j) or olaparib (k). Cell viability was estimated by CellTiter-Glo reagent after ten days drug exposure. Data are mean Surviving Fractions ± SD, *n*=3. Two-way ANOVA with Sídák post hoc test was used to calculate *p* values. (**k,l)** Western blots are shown derived from lysates described in Figure 3m.

**Supplementary Figure 3. Related to Figure 3**

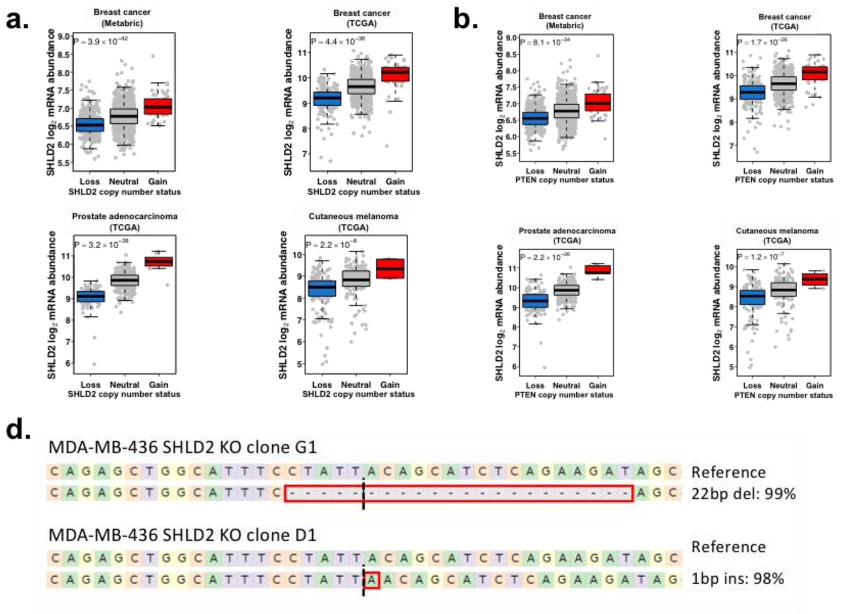


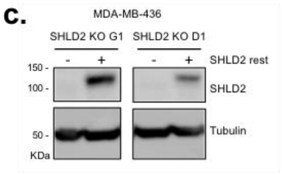


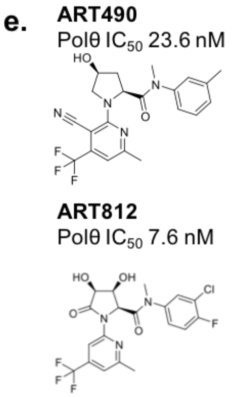


**f.**

| | ART490 | ART558 | ART812 |
|---|---|---|---|
| Biochemical POLQ polymerase $IC_{50}$ (nM) | 23.6 (n= 33) | 7.9 (n=58) | 7.6 (n=62) |
| Cell based MMEJ $IC_{50}$ (nM) | - | 138 (n=2) | 240 (n=12) |
| BRCA-2 DLD-1 CFA $EC_{50}$ (μM) | 2.35 (n= 48) | 1.01 (n=7) | 1.65 (n=5) |
| **Rat Microsomal stability (μL/min/mg)** | **-** | **697** | **50** |
| Rat PK | | | |
| PPB (fraction unbound) | | | 0.05 |
| IV 3 mg/kg | | | |
| Cl (mL/min/kg) | | | 35 |
| T1/2 (hr), Vss (L/kg) | | | 1.3, 2.9 |
| PO 10 mg/kg, 200 mg/kg | | | |
| Free Cmax μM | | | 0.08, 3.3 |
| PO F% | | | 57, nc |

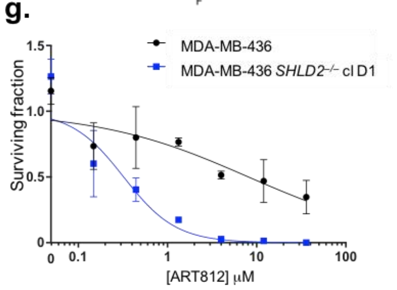


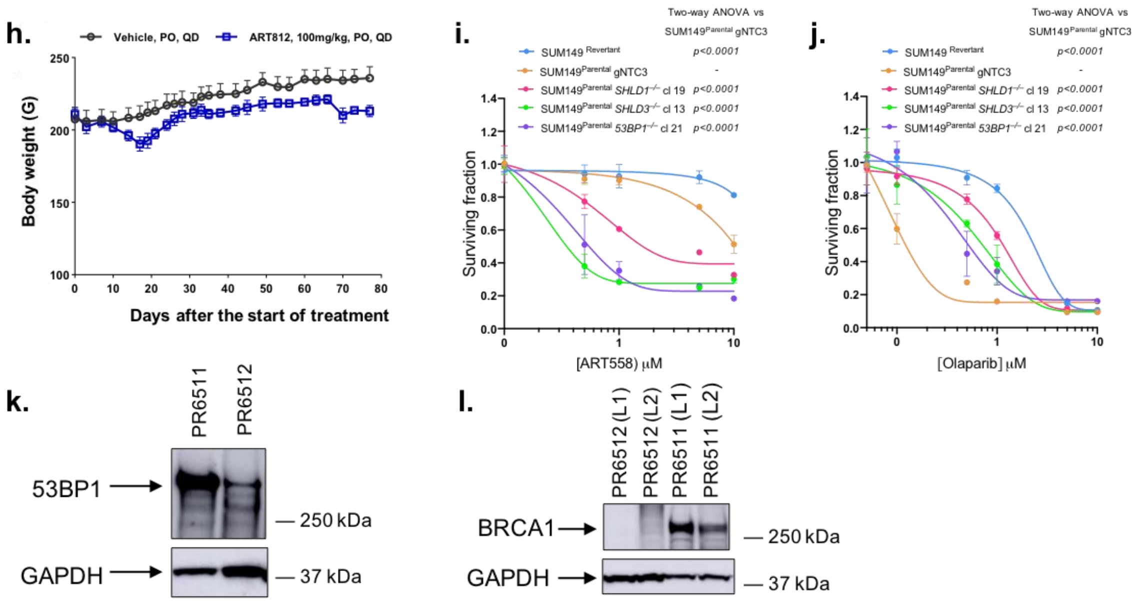


**Figure S3. Defects in the Shieldin complex cause PARP inhibitor resistance but Polθ inhibitor synthetic lethality - Related to figure 3. Figure legend on previous page.**

**Supplementary Figure 4. Related to Figure 4**

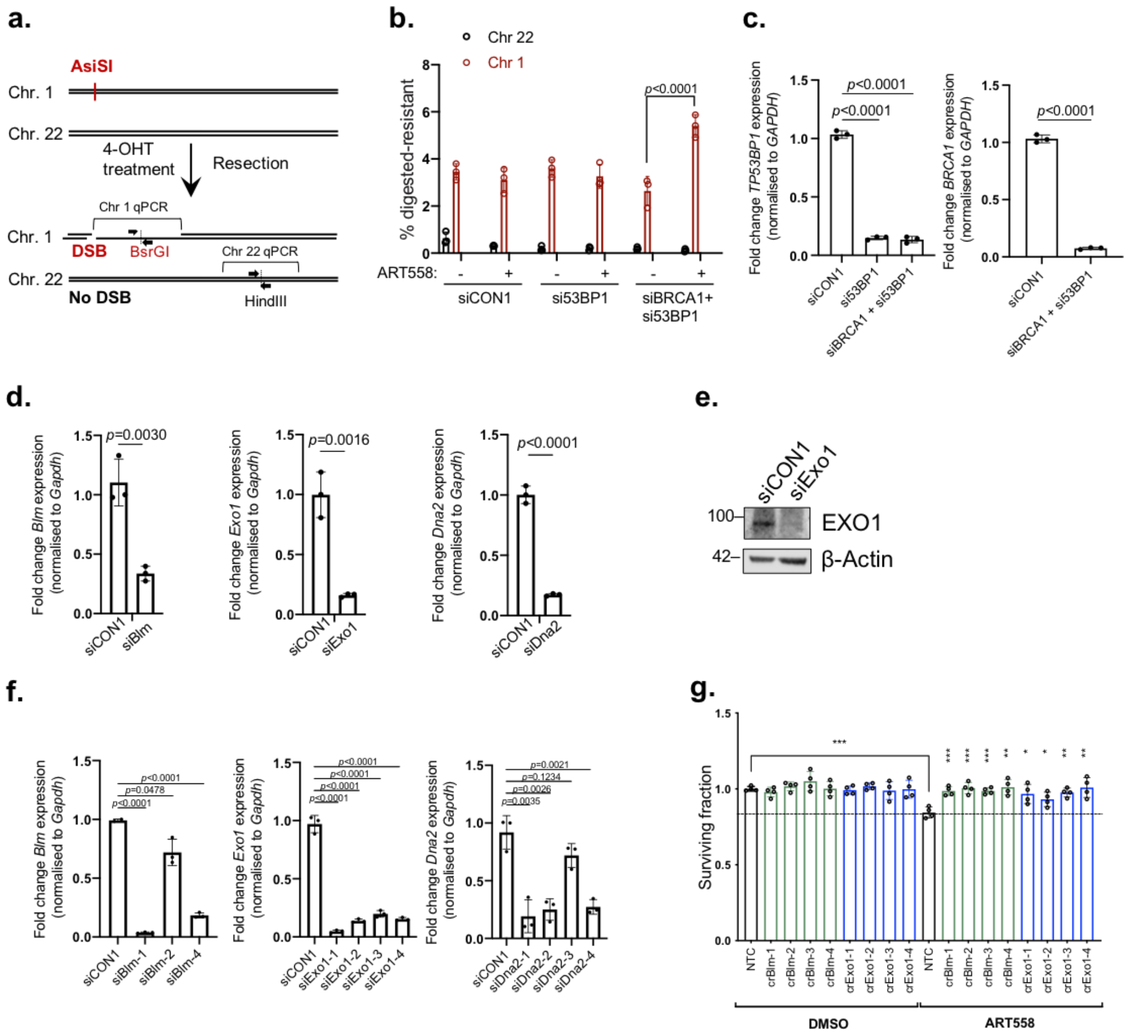


**Supplementary Figure 4. DNA damage-related phenotypes in *Brca1*$^{\Delta 11}$*;Trp53bp1*$^{-/-}$ cells exposed to ART558 - Related to figure 4. (a**) Schematic of the AsiSi DiVa assay in U2OS cells. Design of qPCR primers for measurement of resection at sites adjacent to an AsiSI site located on Chromosome 1. The primers on Chromosome 22 ('No DSB') were used as negative control. The primer pairs are across BsrGI restriction sites. The primer pair for 'No DSB' is across a HindIII restriction site. (**b**) ER-AsiSI U2OS cells were transfected with the different siRNAs, followed by DMSO or ART558 (10μM, 48hrs). Next, cells were treated with 300 nM 4-OHT for 5 h, genomic DNA (gDNA) was extracted and digested or mock digested with BsrGI or HindIII overnight. DNA end resection adjacent to the AsiSi site (335 bp) and No DSB site was measured by qPCR as described in 'Materials and Methods' section. Data are mean ± SD, *n*=3 independent experiments. Two-way ANOVA with Sídák post hoc test was used to calculate p values. (**c**) Bar chart illustrating *TP53BP1* (left) or *BRCA1* (right) mRNA fold change following transfection of ER-AsiSI U2OS cells with a non-targeting siRNA (siCON1) or siRNA pool targeting the indicated gene. mRNA levels were determined by RT-PCR two days after siRNA reverse transfection and normalized to *GAPDH* mRNA levels. Data are mean ± SD, n=3 technical replicates, representative of two independent experiments. Two-tailed Student's t-test was used to calculate *p* value. (**d**) Bar chart illustrating *Exo1, Blm* or *Dna2* mRNA fold change following transfection of *Brca1*$^{\Delta 11}$ *Trp53bp1*$^{-/-}$ MEF cells with a non-targeting siRNA (siCON1) or siRNA pool targeting the indicated gene. mRNA levels were determined by RT-PCR two days after siRNA reverse transfection and normalized to *Gapdh* mRNA levels. Data are mean±SD, n=3 technical replicates, representative of two independent experiments. Two-tailed Student's t-test was used to calculate *p* value. (**e**) Western blot illustrating the knockdown efficiency of siExo1 – Smart Pool in comparison to siCON1. β-Actin was used as a loading control (related to figure 4F). (**f**) Bar chart illustrating *Exo1, Blm* or *Dna2* mRNA fold change following transfection of *Brca1*$^{\Delta 11}$ *Trp53bp1*$^{-/-}$ MEF cells with a non-targeting siRNA (siCON1) or individual siRNAs l targeting the indicated genes. mRNA levels were determined by RT-PCR two days after siRNA reverse transfection

and normalized to *Gapdh* mRNA levels*.* Data are mean ± SD, n=3 technical replicates, representative of two independent experiments. Two-tailed Student's t-test was used to calculate *p* value. (**g**) Column chart illustrating Surviving Fraction of *Brca1*$^{\Delta 11}$*;Trp53bp1*$^{-/-}$ MEFs transfected with the indicated individual crRNA and exposure to DMSO or 5 µM ART558 for 5 days. Surviving fractions are shown relative to median DMSO siCON1. Data are mean ± SD, n=4 independent experiments. Two tailed Student's *t* test was used to calculate *p* values. *=0.0332 **=0.0021, ***=0.0002,

## Supplementary Table 1.

| | Polα $IC_{50}$ (mM) | Polγ $IC_{50}$ (mM) | Polη $IC_{50}$ (mM) | Polν $IC_{50}$ (mM) | DNA binding $IC_{50}$ (mM) |
|---|---|---|---|---|---|
| ART558 | >100 | >100 | >100 | >100 | >100 |

**Pol α, Pol γ, Pol η and Pol ν inhibition elicited by ART558**. IC50 values are shown. For reference ART558 IC50 = 7.9 nM.

**Supplementary Table 2.** siRNA and crRNA used in the study

| | Target | Dharmacon Cat. No. |
|---|---|---|
| Mouse | | |
| | siExo1 SmartPool | M-060591-01-0005 |
| | Exo1 siRNA 1 | D-060591-01-0002 |
| | Exo1 siRNA 2 | D-060591-02-0002 |
| | Exo1 siRNA 3 | D-060591-03-0002 |
| | Exo1 siRNA 4 | D-060591-04-0002 |
| | siBlm SmartPool | M-061987-01-0005 |
| | Blm siRNA 1 | D-061987-01-0002 |
| | Blm siRNA 2 | D-061987-02-0002 |
| | Blm siRNA 4 | D-061987-04-0002 |
| | siDna2 SmartPool | M-062864-01-0005 |
| | Dna2 siRNA 1 | D-062864-01-0002 |
| | Dna2 siRNA 2 | D-062864-02-0002 |
| | Dna2 siRNA 3 | D-062864-03-0002 |
| | Dna2 siRNA 4 | D-062864-04-0002 |
| Human | | |
| | POLQ siRNA 1 | D-015180-01-0005 |
| | POLQ siRNA 2 | D-015180-02-0005 |
| | POLQ siRNA 3 | D-015180-04-0005 |
| | siPOLQ SmartPool | M-015180-01-0005 |
| | siBRCA1 SmartPool | M-003461-02-0005 |
| | siTP53BP1 SmartPool | M-003548-01-0005 |

| | Target | Dharmacon Cat. No. |
|---|---|---|
| Mouse | | |
| | Exo1 crRNA 1 | CM-060591-01-0002 |
| | Exo1 crRNA 2 | CM-060591-02-0002 |
| | Exo1 crRNA 3 | CM-060591-03-0002 |
| | Exo1 crRNA 4 | CM-060591-04-0002 |
| | Blm crRNA 1 | CM-061987-01-0002 |
| | Blm crRNA 2 | CM-061987-02-0002 |
| | Blm crRNA 3 | CM-061987-03-0002 |
| | Blm crRNA 4 | CM-061987-04-0002 |

**Supplementary Table 3.** Primers used in the study

| | Primer Name | Primer Sequence 5'-3' |
|---|---|---|
| **AsiSi DiVa** | | |
| | DSB1-335 FW | GAATCGGATGTATGCGACTGATC |
| | DSB1-335 REV | TTCCAAAGTTATTCCAACCCGAT |
| | NoDSB FW | ATTGGGTATCTGCGTCTAGTGAGG |
| | NoDSB REV | GACTCAATTACATCCCTGCAGCT |
| **Polθ amplification** | Forward | GGG GAC AAG TTT GTA CAA AAA AGC AGG CTT Cat gaa tct tct gcg tcg gag tgg |
| | Reverse | GGG GAC CAC TTT GTA CAA GAA AGC TGG GTA tta cac atc aaa gtc ctt tag ct |
| **PCR-based TMEJ repair assay.** | S1F | CTT ACG TTT GAT TTC CCT GAC TAT ACAG and S2R 5’-AGC AGG GTA GCC AGT CTG AGA TGGG |
| **DNA complex preparation** | Oligonucleotide 1 | GCGGCTGTCATAAG |
| | Oligonucleotide 2 | GCTACATTGACAATGGCATCAAATCTCAGATTG CGTCTTATGACAGCCGCG |
| | Oligonucleotide 3 | CGCGGCTGTCATAAGACGCAATCTGAGATTTGA TGCCATTGTCAATGTAGC |
| | T8 oligonucleotide | TTTCCAATGACAGCCGC |
| | P6 oligonucleotide | GCGGCTGTCATT |

## **Supplementary Table 4.** Small molecule screening data

| Category | Parameter | Description |
|---|---|---|
| Assay | Type of assay | In vitro enzyme assay |
| | Target | PolΘ |
| | Primary measurement | Fluorescent nucleic acid stain for determining double stranded DNA |
| | Key reagents | **Protein**: N-His, C-term FLAG tagged PolΘ protein (amino acids 2-2590) expressed in baculovirus **Buffer:** 25 mM Tris HCl pH 7.5, 12.5 mM NaCl, 0.5 mM $MgCl_2$, 5% glycerol, 0.01% Triton X-100, 0.01% BGG and 1 mM DTT **Primer:** (5’ - GCG GCT GTC ATA AG – 3’):template (5’ – GCT ACA TTG ACA ATG GCA TCA AAT CTC AGA TTG CGT CTT ATG ACA GCC GCG – 3’) duplex (PTD) **Stop reagents:** 4 mM EDTA, 25 mM Tris pH7.5 **Nucleic acid stain:** PicoGreen dye (Invitrogen P7581) |
| | Assay protocol | PicoGreen assay was used to measure the ability of compounds to bind to and inhibit the activity of PolΘ in vitro. N-His, C-term FLAG tagged PolΘ protein (amino acids 2-2590) expressed in baculovirus was purified and stored at -80ºC in aliquots. Assay measurements were performed with 1X buffer comprising 25 mM Tris HCl pH 7.5, 12.5 mM NaCl, 0.5 mM $MgCl_2$, 5% glycerol, 0.01% Triton X-100, 0.01% BGG and 1 mM DTT. Test compounds were prepared by dilution in 100% DMSO and screened at single point concentration of 30 µM dispensing into 384 well micro assay plates (Nunc low volume black) using a Labcyte Echo 550 acoustic dispenser. DMSO concentration was maintained at 1% by adding 0.75% DMSO to the assay buffer. Purified recombinant PolΘ and primer template duplex (PTD) solution (1:1.1 ratio of Primer 5’ - GCG GCT GTC ATA AG – 3’ and Template 5’ – GCT ACA TTG ACA ATG GCA TCA AAT CTC AGA TTG CGT CTT ATG ACA GCC GCG – 3’) were diluted in assay buffer to a 2X working concentration (4 nM PolΘ and 100 nM PTD) with 3 µL of this solution was dispensed into positive control and test wells of the compound plate using a Multidrop Combi. 3. µL of a solution containing PTD only diluted in assay buffer was dispensed into negative control wells of the compound plate using a Multidrop Combi. The assay plates were pre-incubated at room temperature for 30 minutes before addition of 3 µL of 2X working solution of dNTPs (40 µM) (dATP, dCTP, dGTP, dTTP; Sigma D6500, D4635, D4010, T0251) diluted in assay buffer to all wells of the assay plate. The reaction was incubated for 60 minutes at room temperature then stopped by addition of 4ul added of |

| | | |
|---|---|---|
| | | 10mM EDTA, 1:80 Picogreen (Invitrogen P7581) in 25 mM Tris pH7.5 (final concentration of EDTA 4mM and 1:200 Picogreen). After 90 minutes at room temperature. in the dark, fluorescence was read on a BMG Pherastar FS plate reader using 485/520nm module. |
| | Additional comments | |
| Library | Library size | ~165,000 |
| | Library composition | Drug like molecules |
| | Source | Commercial vendors and in house proprietary compounds |
| | Additional comments | |
| Screen | Format | 384 well plate |
| | Concentration(s) tested | 30 µM |
| | Plate controls | 100% inhibited control wells (no enzyme), 0% inhibited wells (full reaction with DMSO), Doxorubicin was used as reference and tested at 1 uM and 0.4 uM on each plate |
| | Reagent/ compound dispensing system | ECHO 550 (Labcyte) acoustic dispenser for compound dispense and Multidrop combi for reagent dispense |
| | Detection instrument and software | BMG PHERAstar and MARS software |
| | Assay validation/QC | Robust Z' >0.5 required to pass each assay plate (unknown criteria for reference compound, +/-20%?) |
| | Correction factors | A Hybrid Median Filtering (HMF) method[1] used for correction, this method corrects systematic errors without affecting the hit selection rate. The corrected data is calculated by the following formula:<br>Corrected Value = (G/L) × Target Value<br>Where: G = median of normalised data for all wells in the plate;  L = calculated local background;  Target value = normalised raw data |
| | Normalization | |
| | Additional comments | |
| Post-HTS analysis | Hit criteria | >50% inhibition at 30 µM |
| | Hit rate | 0.72% |
| | Additional assay(s) | **Interference assay**: The PolΘ enzyme reaction is run separate from the compounds. The reaction is stopped and the Picogreen dye added before then dispensing the completed reaction into assay plates containing compound.<br>**Orthogonal assay**: Differential Scanning Fluorimetry (DSF) was used as an orthogonal technique to validate the hits. |
| | Confirmation of hit purity and structure | Hits were resynthesized, structure and purity were confirmed using LCMS and 1H NMR |
| | Additional comments | |
