# Supplementary material for: Polθ inhibitors elicit BRCA-gene synthetic lethality and target PARP inhibitor resistance
Source: Nat Commun. 2021 Jun 17;12:3636. doi: 10.1038/s41467-021-23463-8 (PMC8211653; doi:10.1038/s41467-021-23463-8)
Supplement: Supplementary file 3 — Description of Additional Supplementary Files [file 41467_2021_23463_MOESM3_ESM.docx]

Description of Additional Supplementary Files

Title: Supplementary Data 1

Description: siRNA screen DE Z scores
