# Supplementary material for: Polθ inhibitors elicit BRCA-gene synthetic lethality and target PARP inhibitor resistance
Source: Nat Commun. 2021 Jun 17;12:3636. doi: 10.1038/s41467-021-23463-8 (PMC8211653; doi:10.1038/s41467-021-23463-8)
Supplement: Supplementary file 5 — Reporting Summary [file 41467_2021_23463_MOESM5_ESM.pdf]

nature research

Corresponding author(s): Christopher J. Lord, Stephen J Pettitt, Graeme Smith

Last updated by author(s): Apr 26, 2021

Reporting Summary

Nature Research wishes to improve the reproducibility of the work that we publish. This form provides structure for consistency and transparency in reporting. For further information on Nature Research policies, see our Editorial Policies and the Editorial Policy Checklist.

Statistics

For all statistical analyses, confirm that the following items are present in the figure legend, table legend, main text, or Methods section.

n/a	Confirmed	
☐	☒	The exact sample size (n) for each experimental group/condition, given as a discrete number and unit of measurement
☐	☒	A statement on whether measurements were taken from distinct samples or whether the same sample was measured repeatedly
☐	☒	The statistical test(s) used AND whether they are one- or two-sided<br>*Only common tests should be described solely by name; describe more complex techniques in the Methods section.*
☒	☐	A description of all covariates tested
☒	☐	A description of any assumptions or corrections, such as tests of normality and adjustment for multiple comparisons
☐	☒	A full description of the statistical parameters including central tendency (e.g. means) or other basic estimates (e.g. regression coefficient) AND variation (e.g. standard deviation) or associated estimates of uncertainty (e.g. confidence intervals)
☐	☒	For null hypothesis testing, the test statistic (e.g. F, t, r) with confidence intervals, effect sizes, degrees of freedom and P value noted<br>*Give P values as exact values whenever suitable.*
☒	☐	For Bayesian analysis, information on the choice of priors and Markov chain Monte Carlo settings
☒	☐	For hierarchical and complex designs, identification of the appropriate level for tests and full reporting of outcomes
☒	☐	Estimates of effect sizes (e.g. Cohen's d, Pearson's r), indicating how they were calculated

Our web collection on statistics for biologists contains articles on many of the points above.

Software and code

Policy information about availability of computer code

Data collection	IncuCyte® S3 (v6.2.9200.0) ; Microsoft Excel (v16.16.26); Quant Studio Software (v1.7.1); ImageStudio (v5.2); SlideBook (v6.0.16); ViiA™ 7; Andor iQ3; SkanIt Microplate Reader Software
Data analysis	GraphPad Prism (v8.4.2); Microsoft Office Excel (v16.16.26); MetaExpress (MolDev); SlideBook (v6.0.16); ImageJ (v2.0.0); RStudio; ViiA™ 7; MARS (BMG Labtech); Phoenix WinNonlin 6.3

For manuscripts utilizing custom algorithms or software that are central to the research but not yet described in published literature, software must be made available to editors and reviewers. We strongly encourage code deposition in a community repository (e.g. GitHub). See the Nature Research guidelines for submitting code & software for further information.

Data

Policy information about availability of data

All manuscripts must include a data availability statement. This statement should provide the following information, where applicable:
- Accession codes, unique identifiers, or web links for publicly available datasets
- A list of figures that have associated raw data
- A description of any restrictions on data availability

Publicly available data used in this paper were obtained from cbioportal.org, February 2021 : Breast Cancer (Metabric), Breast Cancer (TCGA), Prostate adenocarcinoma (TCGA), Cutaneous melanoma (TCGA) and Ovarian cancer (TCGA). https://www.cbioportal.org/datasets
In supplementary figure 2d we used the publicly available CRISPR-Cas9 screen data (https://doi.org/10.1038/s41586-018-0291-z Zimmermann, M. et al. Nature 2018).

Field-specific reporting

Please select the one below that is the best fit for your research. If you are not sure, read the appropriate sections before making your selection.

- [x] Life sciences
- [ ] Behavioural & social sciences
- [ ] Ecological, evolutionary & environmental sciences

For a reference copy of the document with all sections, see nature.com/documents/nr-reporting-summary-flat.pdf

Life sciences study design

All studies must disclose on these points even when the disclosure is negative.

Sample size	No statistical methods were used to pre-determine sample size. Sample sizes (at least 3 independent experimental replicates in most experiments, unless indicated otherwise) were chosen based on the standard practices of the field.
Data exclusions	No data was excluded from this study.
Replication	Data was successfully replicated in at least 2 independent experiments (details of the replication are included in figure legends of Individual experiment).
Randomization	Animals were randomized by individual tumor volume when mean tumor volume reached 250-350 mm3. Animals were randomized into three groups of 8 animals each using Vivo Manager® software (Biosystemes, France). Homogeneity between groups was tested by an analysis of variance (ANOVA).<br>Randomization is not applicable for in vitro experiments as genetic knockouts or wild type cells with different treatments cannot be randomized.
Blinding	Groups were randomized in a blinded fashion. The Technicians performing the experiments were unaware of the agents used in each treatment group.

Reporting for specific materials, systems and methods

We require information from authors about some types of materials, experimental systems and methods used in many studies. Here, indicate whether each material, system or method listed is relevant to your study. If you are not sure if a list item applies to your research, read the appropriate section before selecting a response.

Materials & experimental systems

n/a	Involved in the study	
☐	☒	Antibodies
☐	☒	Eukaryotic cell lines
☒	☐	Palaeontology and archaeology
☐	☒	Animals and other organisms
☒	☐	Human research participants
☒	☐	Clinical data
☒	☐	Dual use research of concern

Methods

n/a	Involved in the study	
☒	☐	ChIP-seq
☒	☐	Flow cytometry
☒	☐	MRI-based neuroimaging

Antibodies

Antibodies used

Primary antibodies used in Western blot studies:
TP53BP1 - Bethyl A300-272A
Vinculin - Santa Cruz sc-73614
Beta-actin - Cell signaling technologies 13E5
POLQ - Gift from J.S. Hoffman
gH2Ax - Merck Millipore 05-636
BRCA1 - Santa Cruz sc-6954
53BP1 - Antibodies-online ABIN1724821
GAPDH - Cell signaling technologies - 3683S
Tubulin - Abcam ab7291
SHLD2 - generated for Artios Pharma by CRB
EXO1 - Bethyl A302-640A

Primary antibodies used in immunofluorescence studies
pRPA (S4/S8) - Bethyl A300-245A
gH2Ax - Merck Millipore 05-636
BrdU - GE Healthcare RPN202

	RPA - Abcam ab2175<br>PCNA - Santa Cruz sc7907<br><br>Secondary antibodies used in Western blot studies:<br>IRDye® 680RD Donkey anti-Mouse IgG - Li-Cor 926-68072<br>IRDye® 800CW Goat anti-Rabbit IgG - Li-Cor 926-32211<br>Goat anti-Rabbit IgG HRP - Sigma A9169-2ML<br>Goat anti-mouse IgG HRP - Invitrogen 31430-2ML<br><br>Secondary antibodies used in immunofluorescence studies<br>Alexa Fluor 555–conjugated mouse - Thermo Fisher Scientific A-21422<br>Alexa Fluor 488–conjugated rabbit - Thermo Fisher Scientific A-11034
Validation	TP53BP1 - Bethyl A300-272A Validation stated on supplier's website https://www.bethyl.com/product/A300-272A/53BP1+Antibo;<br>Vinculin - Santa Cruz sc-73614 Validation stated on supplier's website https://www.scbt.com/p/vinculin-antibody-7f9;<br>Beta-actin - Cell signaling technologies 13E5 Validation stated on supplier's website https://www.cellsignal.co.uk/datasheet.jsp?productId=4970&images=0<br>POLQ - Gift from J.S. Hoffman Validation stated in: Fernandez-Vidal, A., Guitton-Sert, L., Cadoret, J. et al. A role for DNA polymerase θ in the timing of DNA replication. Nat Commun 5, 4285 (2014);<br>pRPA (S4/S8) - Bethyl A300-245A Validation stated on supplier's website https://www.bethyl.com/product/A300-245A/Phospho+RPA32+(S4+S8)+Antibody;<br>gH2Ax - Merck Millipore 05-636 Validation stated on supplier's website https://www.merckmillipore.com/GB/en/product/Anti-phospho-Histone-H2A.X-Ser139-Antibody-clone-JBW301,MM_NF-05-636;<br>BrdU - GE Healthcare RPN202 Validation stated on supplier's website https://www.sigmaaldrich.com/catalog/product/sigma/gerpn202?lang=en&region=GB;<br>RPA - Abcam ab2175 Validation stated on supplier's website https://www.abcam.com/rpa32rpa2-antibody-9h8-ab2175.html;<br>PCNA - Santa Cruz sc7907 Validation stated on supplier's website https://www.scbt.com/p/pcna-antibody-fl-261;<br>BRCA1 - Santa Cruz sc-6954 https://datasheets.scbt.com/sc-6954.pdf<br>53BP1 - Antibodies-online ABIN1724821 Validation stated on supplier's website https://www.antibodies-online.com/productsheets/ABIN1724821.pdf<br>GAPDH - Cell signaling technologies - 3683S Validation stated on supplier's website https://www.cellsignal.com/datasheet.jsp?productId=3683&images=1<br>Tubulin - abcam ab7291 Validation stated on supplier's website https://www.abcam.com/alpha-tubulin-antibody-dm1a-loading-control-ab7291.html<br>SHLD2 - generated for Artios Pharma by CRB and has been validated internally. Data can be supplied on request and / or added to supplementary info.<br>EXO1 - Bethyl A302-640A Validation stated on supplier's website https://www.bethyl.com/product/pdf/A302-640A.pdf

Eukaryotic cell lines

Policy information about cell lines

Cell line source(s)	HEK-293 - source: ATCC<br>SUM149 cells - source: Asterand<br>CAL51 cells - source: DSMZ,<br>COV362 - source: ECACC<br>MDA-MB-436 - source: ATCC<br>MDA-MB-436 SHLD2 clones were generated in this study.<br>DLD1 BRCA2 Wild-type and DLD1 BRCA2–/– cells - source: Horizon Discovery Inc.<br>CAPAN1Parental - source: ATCC<br>U2OS Flp-In T-rex - Flp-In T-rex system source: Invitrogen and the host U2OS cells were a gift from D. Durocher<br>YFP-Polq FlpIN Trex U2OS cells were generated in this study<br>RPE1 TP53–/– BRCA1Wild-Type and RPE1 TP53–/– BRCA1–/– cells were a gift from D. Durocher - original source ATCC<br>Mouse embryonic fibroblasts (MEFs) were generated from E13.5 embryos and were a gift from A. Nussenzweig.<br>129/Ola-derived IB10 mouse embryonic stem cells were a gift from M. Tijsterman and were described in Zelensky, A.N., Schimmel, J., Kool, H. et al. Inactivation of Pol θ and C-NHEJ eliminates off-target integration of exogenous DNA. Nat Commun 8, 66 (2017).<br>Mouse ID8 cells were described in Walton J.B. et al. Sci Rep 7; 16827 (2017)
Authentication	HEK293, SUM149, CAL51, COV362, MDA-MB-436, CAPAN1 cell lines were authenticated using STR DNA profiling using the Geneprint 10 system, and involves the amplification of nine human loci, including the ASN-0002 loci (TH01, TPOX, vWA, Amelogenin, CSF1PO, D16S539, D7S820, D13S317 and D5S818) as well as D21S11.<br><br>The mouse embryonic, ID8 lines and DLD1 BRCA2 Wild-type and DLD1 BRCA2–/– were validated using functional assays (PARP inhibitor sensitivity, RAD51 focus formation after DNA damage and immunoblotting for 53BP1, BRCA1 and BRCA2 expression, respectively).<br><br>YFP-Polq FlpIN Trex U2OS cells were validated by immunoblotting for the YFP and Polq components of the transgene.
Mycoplasma contamination	All the cell lines used were mycoplasma negative.

Commonly misidentified lines (See ICLAC register)	No commonly misidentified cell lines were used in this study.

Animals and other organisms

Policy information about studies involving animals; ARRIVE guidelines recommended for reporting animal research

Laboratory animals

Female SRG OncoRats (SD-Rag2tm2Hera Il2rgtm1Hera/HeraArc), 5-7 weeks old at reception, were obtained from Charles River. Expansion of the PDXO model was carried out in NSG hosts by Crown BioScience following their optimised protocols (https://www.crownbio.com/oncology/ex-vivo-services/3d-assays/).

Housing conditions:

Animals were maintained in housing rooms under controlled environmental conditions:
Temperature: 20 ± 2°C,
Humidity 35 ± 10%,
Photoperiod (12h light/12h dark),
HEPA filtered air,
15 air exchanges per hour with no recirculation.
Animal enclosures provided sterile and adequate space with bedding material, food and water, environmental and social enrichment (group housing) as described:
Top filter polycarbonate Eurostandard Type III or IV cages,
Beta Chip® bedding (Northeastern Products, North America),
25 kGy Irradiated diet (ref: 2916, Envigo, North America),
Osmotic water,
Environmental enrichment (Tube PVC ½'sterile, Canadian Tire, Canada).

Wild animals

No wild animals were used in the study.

Field-collected samples

No field collected samples were used in the study.

Ethics oversight

Animal housing and experimental procedures were conducted according to the Guide for the care and use of experimental animals issued by the Canadian Council on Animal Care and the National Research Council Guide for the Care and Use of Laboratory Animals

Note that full information on the approval of the study protocol must also be provided in the manuscript.
